# Effect of astaxanthin supplementation on female fertility and reproductive outcomes: a systematic review and meta-analysis of clinical and animal studies

**DOI:** 10.1186/s13048-024-01472-7

**Published:** 2024-08-10

**Authors:** Arezoo Maleki-Hajiagha, Anahid Shafie, Khadije Maajani, Fardin Amidi

**Affiliations:** 1 Department of Anatomy, School of Medicine, Tehran University of Medical Sciences, Pour Sina St, Tehran, 1416753955 Iran; 2 Students’ Scientific Research Center (SSRC), Tehran University of Medical Sciences, Tehran, Iran; 3 Department of Epidemiology and Biostatistics, School of Public Health, Tehran University of Medical Sciences, Tehran, Iran; 4 Department of Infertility, Yas Hospital, Tehran University of Medical Sciences, Tehran, Iran

**Keywords:** Astaxanthin, Infertility, Fertility, Reproduction, Oxidative stress, Assisted reproductive technology

## Abstract

**Context:**

Oxidative stress (OS) plays a harmful role in female reproduction and fertility. Several studies explored various dietary interventions and antioxidant supplements, such as astaxanthin (AST), to mitigate the adverse effects of OS on female fertility. Ameliorative effects of AST on female fertility and the redox status of reproductive organs have been shown in several animal and clinical studies.

**Objectives:**

The main objective of present systematic review and meta-analysis of both animal and clinical studies was to provide a comprehensive overview of the current evidence on the effects of AST on female fertility and reproductive outcomes. The effect of AST on redox status, inflammatory and apoptotic markers in reproductive organs were included as the secondary outcomes.

**Data sources:**

We systematically searched electronic databases including PubMed, Scopus, and Web of Science, until January 1, 2024, using specified search terms related to AST, female reproductive performance, and infertility, considering the diverse synonyms found in the literature for interventional studies that compared oral AST supplementation with placebo or control in human or animal models.

**Data extraction:**

Two independent reviewers extracted data on study characteristics, outcomes, and risk of bias. We pooled the results using random-effects models and assessed the heterogeneity and quality of evidence. We descriptively reported the data from animal models, as meta-analysis was not possible.

**Data analysis:**

The meta-analysis of clinical trials showed that AST significantly increased the oocyte maturation rate (MD: 8.40, 95% CI: 4.57 to 12.23, I^2^: 0%) and the total antioxidant capacity levels in the follicular fluid (MD: 0.04, 95% CI: 0.02 to 0.06, I^2^: 0%). The other ART and pregnancy outcomes and redox status markers did not show statistically significant changes. The animal studies reported ameliorative effects of AST on redox status, inflammation, apoptosis, and ovarian tissue histomorphology.

**Conclusion:**

This systematic review shows that AST supplementation may improve assisted reproductive technology outcomes by enhancing oocyte quality and reducing OS in the reproductive organs. However, the evidence is limited by the heterogeneity, risk of bias, and small sample size of the included studies.

**Graphical Abstract:**

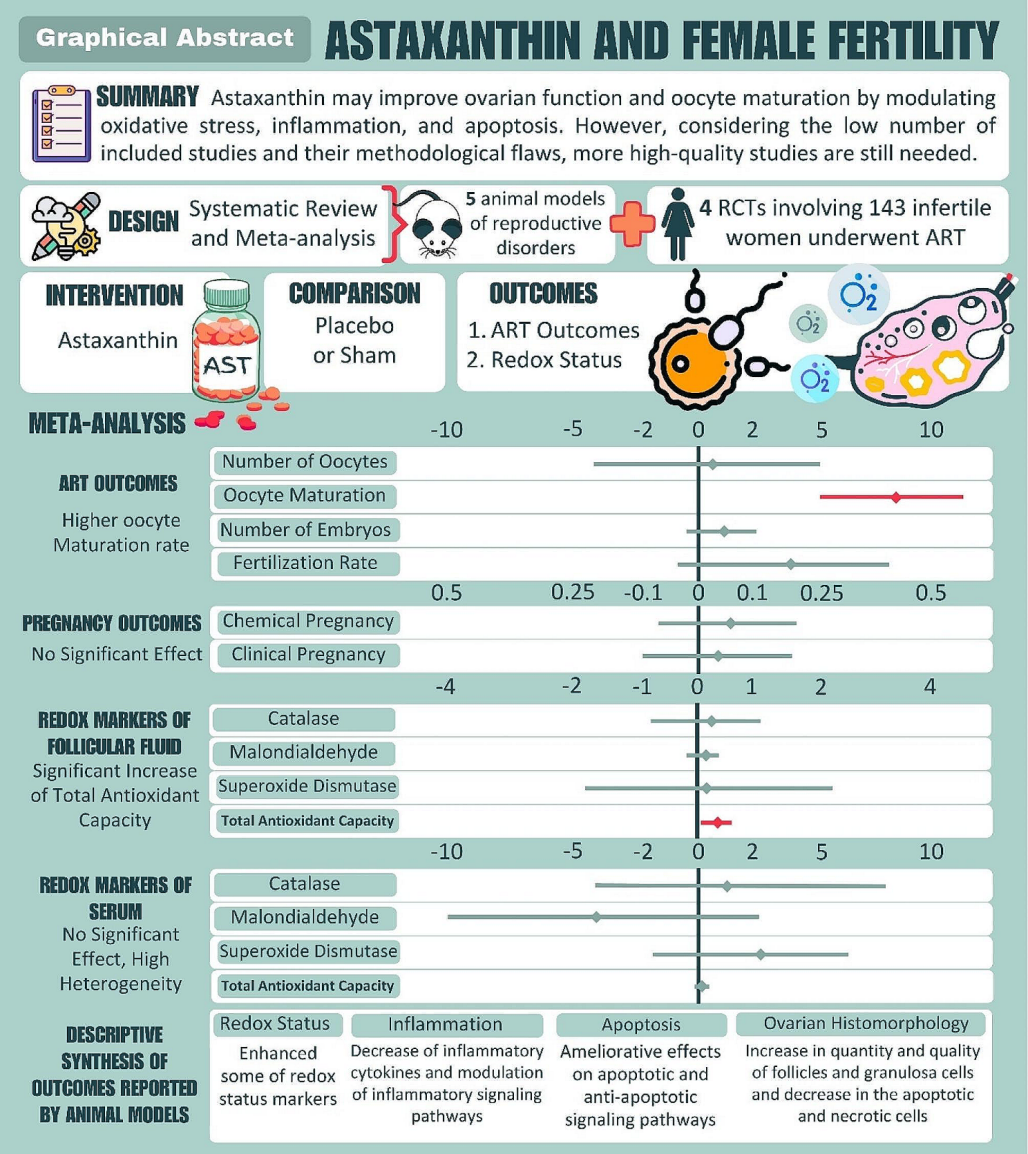

**Supplementary Information:**

The online version contains supplementary material available at 10.1186/s13048-024-01472-7.

## Introduction

Infertility, which according to the World Health Organization definition is described as “the failure to achieve a clinical pregnancy after 12 months or more of regular unprotected sexual intercourse” [1], impacts approximately 1 in 6 couples globally [2]. Close to 50% of infertility cases can be traced back to female factors, as outlined in previous studies [3]. These factors span a spectrum, ranging from hormonal imbalances and anatomical abnormalities to the inevitable decline in oocyte quantity and quality associated with aging. Despite these known factors, a considerable portion of infertility cases remains unexplained.

Oxidative stress (OS) plays a deleterious role in female reproduction and fertility and occurs when the body produces free radicals, including reactive oxygen species (ROS), exceeding its antioxidant defense capacity [4]. Physiological levels of ROS are necessary for normal reproductive system performance, while their overabundance can exert detrimental effects on fertility [5]. OS can damage the DNA and mitochondria of ovarian follicles and oocytes and compromise their viability and fertilization potential [6, 7]. Also, a growing body of evidence has linked OS to the etiology of various female reproductive disorders, including polycystic ovary syndrome (PCOS), endometriosis, and diminished ovarian reserve [8–10]. Accordingly, several studies explored various dietary interventions and antioxidant supplements to mitigate the adverse effects of OS on female reproductive performance and fertility.

Among various antioxidants, Astaxanthin (3,3′-dihydroxy-β, β′-carotene-4,4′-dione, (AST)), a xanthophyll carotenoid [11] widely recognized as the “king of antioxidants”, stands out as a compelling choice, warranting further exploration and scrutiny. AST’s antioxidant potential has been shown to be superior to vitamin C, vitamin E, coenzyme Q10, and alpha-lipoic acid, according to reports [12]. Numerous investigations have consistently demonstrated the multifaceted benefits of AST, encompassing its antioxidant, anti-inflammatory, and anti-apoptotic properties by engaging various molecules and signaling pathways [13]. AST enhances antioxidant defense via activating the nuclear factor erythroid 2-related factor 2/heme oxygenase-1 (Nrf2/HO-1) signaling pathway, and consequently neutralizes free radicals and safeguards cell membranes [14]. Nrf2/HO-1 activation by AST promotes oocyte maturation rate (OMR) and high-quality embryo yield in infertile women undergoing assisted reproductive technology (ART) [15]. Additionally, considering the modulatory effect of AST on the nuclear factor kappa B (NF-κB) signaling, it can regulate inflammatory responses which are responsible for the pathogenesis of various infertility-associated disorders like endometriosis and PCOS [16, 17]. Moreover, AST potentially promotes fertility by mitigating apoptosis of the granulosa cells (GCs) through phosphoinositide 3-kinase/protein kinase B (PI3K/Akt) signaling pathway [18].

While the detrimental impact of OS on female fertility is recognized, and the potential benefits of AST as a powerful antioxidant have been explored, there remains a significant gap in the literature: the absence of a comprehensive review that synthesizes the disparate findings. This gap hinders the ability to draw definitive conclusions about AST’s role and mechanism of action in improving female reproductive health and its therapeutic value in treating infertility. Our study aims to bridge this gap by providing a systematic review that not only consolidates evidence from both animal and clinical studies but also critically examines the methodological diversity, offering recommendations to standardize future research in this domain.

## Materials and methods

We performed a systematic review and meta-analysis in accordance with a predetermined protocol aligned with the guidelines of the Cochrane Collaboration. We aimed to review the existing evidence on the effect of AST compared to placebo or blank control on fertility and reproductive outcomes in human or animal models. We developed the study question according to the PICO framework, which specifies the population, intervention, comparison, and outcome of interest.

### Reporting standards

We adhered to the guidelines outlined in the Preferred Reporting Items for Systematic Reviews and Meta-Analyses (PRISMA) statement for the execution and documentation of systematic reviews. The PRISMA checklist is shown in supplementary file S1.

### Criteria for considering studies for this review

We applied the following inclusion and exclusion criteria to select the studies for our systematic review:

### Inclusion criteria

#### Study type

Interventional design in both laboratory and clinical settings.

#### Study design

For clinical settings, only randomized controlled trials with either parallel or cross-over design. For laboratory settings, only studies that used target models or diseases of reproductive related disorders or pathologies, such as PCOS, endometriosis, aging, or experimental ovarian injuries (induced by ischemia-reperfusion or toxic agents).

#### Intervention and control

Oral AST supplementation in any dosage or duration compared to placebo in clinical studies. Oral consumption or intraperitoneal injection of AST compared to negative, positive, or sham control groups in animal studies.

#### Outcomes of interest

Our review systematically included clinical and animal studies that reported effect of AST supplementation on different reproductive and fertility outcomes. Additionally, as a secondary objective, we reviewed studies that investigated the primary cellular and molecular mechanisms of action of AST on reproductive organs. This included an assessment of redox status markers, inflammation markers, and apoptosis markers in women or female animal models with reproductive disorders or infertility. Our inclusive approach aimed to provide a comprehensive analysis of AST’s effects on reproductive health, thereby encompassing a broad spectrum of biological pathways.

### Exclusion criteria

We excluded studies if:

- They had a non-experimental design (case studies, case series, cross-sectional, case-control, cohort, and other retrospective studies).
- They had a quasi-randomized design (non-randomized or uncontrolled).
- They enrolled transgenic and ex vivo animal models.
- They harvested genitourinary tissues other than ovaries, such as the uterus, fallopian tubes, vagina, or any other organs, such as the liver.
- They isolated and cultured cells other than oocyte, GCs, and theca cells, such as endometrial cells, trophoblastic cells, or different types of cell lines, such as KGN.
- They used non-mammalian animal models.
- They had an in-vitro design and used AST as a culture or cryopreservation medium additive.
- Precise details regarding the research method or results couldn’t be extracted.
- They were exclusively presented in the form of abstracts, ongoing clinical studies, review articles, letters to the editor, or editorials.

## Search methods for identification of studies

We systematically searched electronic databases, including PubMed, Scopus, and Web of Science, until January 1, 2024, using specified search terms related to AST, female reproductive performance, and infertility, considering the diverse synonyms found in the literature. Additionally, we examined citation lists of relevant publications, review articles and included studies for further pertinent research. Following the Cochrane Collaboration guidelines, we explored grey literature through gray literature databases and unpublished trials via the Open Grey database and clinical trials registration databases. Detailed search information is accessible in supplementary file S2.

## Data collection and analysis

### Selection of studies

The search results of different databases were combined, and duplicated articles were removed. All search results were reviewed by two independent reviewers (A.MH. and A.S.) by screening the title and abstract, followed by a full-text review. Disagreements were settled by discussion and third-party opinion (K.M.).

### Data extraction and management

Two reviewers (A.MH. and A.S.) independently extracted the following data from each eligible study using a predefined, standardized, electronic data extraction form. Disagreements were settled by discussion and the supervisor’s opinion (K.M.).

#### For clinical studies

Author’s name, year of publication, study location, study population, baseline characteristics of study participants, AST dosage, duration and route of administration, study outcomes, and outcome measures.

#### For animal models

Author’s name, year of publication, study location, animal species and breed, animals age, study design, model and method of model development, study groups and sample size in each group, study outcomes and outcome measures, AST dosage, duration, administration route and dissolvent, and signaling pathways evaluated by study.

### Assessment of risk of bias in included studies

Two reviewers (A.MH. and A.S.) independently reviewed full text of included studies and assessed the risk of bias for the included studies. We used the RoB 2 tool [19] for clinical randomized trials and the SYRCLE’s tool [20] for animal interventional studies. These tools are based on the criteria recommended by the Cochrane Handbook for Systematic Reviews of Interventions. The RoB 2 tool is designed to assess the risk of bias in randomized trials included in systematic reviews. The SYRCLE’s risk of bias tool is specifically developed for animal intervention studies. It is based on the Cochrane RoB tool but has been adjusted to address aspects of bias that play a specific role in animal studies. Both of these tools include signaling questions to facilitate judgment and covers domains such as selection bias, performance bias, detection bias, attrition bias, reporting bias, and other biases. The assessment was conducted in several stages:

#### Initial training

Both reviewers underwent a training session to familiarize themselves with the criteria of the RoB 2 and SYRCLE’s tools. They independently assessed a set of training studies and compared their evaluations to ensure consistency in their understanding and application of the assessment criteria.

#### Independent assessment

Each reviewer independently assessed the risk of bias in the included studies. The assessment covered the following domains:

##### Selection bias

Evaluated based on the adequacy of random sequence generation and allocation concealment. Studies were considered at low risk if they described a random and adequately concealed allocation process.

##### Performance bias

Assessed by examining blinding of participants and personnel. Studies that did not blind participants or personnel were rated at high risk.

##### Detection bias

Determined by the blinding of outcome assessment. Studies were judged at low risk if the outcome assessors were blinded to the intervention.

##### Attrition bias

Based on the completeness of outcome data. Studies with high dropout rates or incomplete outcome data were considered at high risk.

##### Reporting bias

Identified through selective reporting. Studies were at high risk if they did not include pre-specified outcomes or reported results selectively.

##### Other sources of bias

Examined additional factors such as sample size calculation and funding sources.

#### Consensus meeting

In cases of discrepancy, the reviewers discussed their assessments to reach a consensus. If no agreement could be reached, a third reviewer (K.M.) was consulted to make a final decision.

#### Risk of bias reporting

The results of the risk of bias assessment were summarized in figures and tables (Figure 2 and Table 3), with each study receiving a judgment of ‘low risk,’ ‘high risk,’ or ‘unclear risk’ for each domain. Additional tables also included notes on the rationale for each judgment (supplementary file S4 and S5).

### Overlapping authorships

We acknowledge that one of our authors (F.A.) have co-authored some of the studies that are included in our review. This may create a potential or perceived conflict of interest for our review team. To address this issue, we ensured that the reviewer who is a co-author of some of the included studies was not involved in screening, data extraction, quality assessment, and synthesis of that study. We also documented and justified any decisions or judgments that we made in relation to the inclusion or exclusion of studies, the extraction or analysis of data, and the interpretation or presentation of findings.

### Study outcomes

#### Primary outcomes

For clinical studies, our meta-analysis primarily focused on ART and pregnancy outcomes, including the total number of oocytes and embryos, OMR, fertilization rate, chemical pregnancy rate, clinical pregnancy rate, miscarriage rate, and live birth rate were selected. Definitions for these outcomes are provided below to ensure consistency and clarity across studies.

OMR is defined as the number of mature oocytes (oocytes advanced from Germinal Vesicle and prophase I to metaphase II of meiosis divisions) per total number of retrieved oocytes. The fertilization rate is defined as the number of fertilized oocytes (confirmed by visualization of two pronuclei, either followed by spermatozoon intracytoplasmic injection or conventional In-vitro Fertilization) per total number of microinjected (or spermatozoon co-incubated) mature oocytes. The chemical pregnancy rate is defined as the number of chemical pregnancies (diagnosed only by the detection of beta Human Chorionic Gonadotropin in serum or urine) divided by the number of embryo transfer cycles. The Clinical pregnancy rate is defined as the number of clinical pregnancies (diagnosed by ultrasonographic visualization of one or more gestational sacs, definitive clinical signs of pregnancy, and also clinically documented ectopic pregnancy) divided by the number of embryo transfer cycles [1].

For animal model studies, indicators of reproductive performance and fertility of mammalians, including fertility, prolificacy (the number of offspring produced per mating or per birth), offspring survival rate, sexual maturity (the age at which an animal becomes capable of reproducing), reproductive lifespan (the duration of reproductive activity were planned to be reviewed. Indicators of reproductive performance in rodents (rats and mice) were estrous cycle length, gestation length, litter size, litter weight, birth weight, and weaning age.

#### Secondary outcomes

In addition to ART and pregnancy outcomes, our systematic review aimed to extract data on AST’s cellular mechanisms of action in reproductive disorders. Therefore, we assessed studies regarding redox status, inflammation, and apoptosis markers in ovarian tissue, follicular fluid (FF), or serum in women or female animal models with reproductive disorders or infertility, as these represent the main documented mechanistic pathways influenced by AST [21]. These markers included reactive oxygen species (ROS) such as superoxide, hydrogen peroxide, and hydroxyl radical; antioxidant molecules or enzymes that can scavenge ROS and prevent or repair oxidative damage, such as glutathione (GSH), superoxide dismutase (SOD), catalase (CAT), and total antioxidant capacity (TAC); oxidative products that reflect the extent of oxidative damage, such as malondialdehyde (MDA), protein carbonyls, and thiobarbituric acid reactive substances (TBARS); inflammatory cytokines like interleukins (IL1ß, IL2, IL6, IL8, etc.), tumor necrosis factor-alpha (TNF-α), interferon gamma (IFNγ), etc.; and apoptosis markers like caspase-3, PARP, Bcl-2, etc.

### Measures of treatment effect

We planned to collect dichotomous data for rates of chemical pregnancy, clinical pregnancy, live birth, and miscarriage and for the continuous data like total number of retrieved oocytes, OMR, total number of embryos, and fertilization rate and redox status markers measurements we collected mean differences (MDs) and the associated standard deviations (SDs). We performed two steps of statistical analysis to examine the effect of AST and placebo on the ART outcomes and redox status. We used Review Manager software (Version 5.4, The Cochrane Collaboration, 2020) and STATA software (Version 11) for the analysis.

#### Meta-analysis of clinical trials

We used a random-effects model to calculate the pooled mean difference (MD) with 95% confidence interval (CI) for the continuous outcomes, such as redox status markers in FF and serum. We used the metaprop command in STATA to pool the chemical and clinical pregnancy rates as proportions, with exact binomial and score-test-based confidence intervals for proportions near boundaries (near 0 or 100%). We also calculated the risk difference (RD) and risk ratio (RR) with corresponding 95% confidence intervals (CIs) for each endpoint in the intervention versus placebo groups. The results were displayed on forest plots. We statistically analyzed the heterogeneity using the χ^2^ test and quantified the degree of heterogeneity with the I^2^ statistic (0–50%: mild to moderate heterogeneity; above 50%: considerable heterogeneity). We planned to assess publication bias using funnel plots and Egger’s regression test, contingent upon having a sufficient number of studies in each meta-analysis.

#### Descriptive synthesis of animal models

We did not perform a meta-analysis of the animal models because none of them reported the eligible fertility and reproductive outcomes of this review and they just reported some qualitative reproductive outcomes like ovarian histopathological characteristics. Moreover, each study used different methods for investigating redox status markers. Therefore, we summarized the data of animal studies descriptively, to present the main results and trends.

## Results

### Literature search and characteristics of included studies

**Fig. 1:**
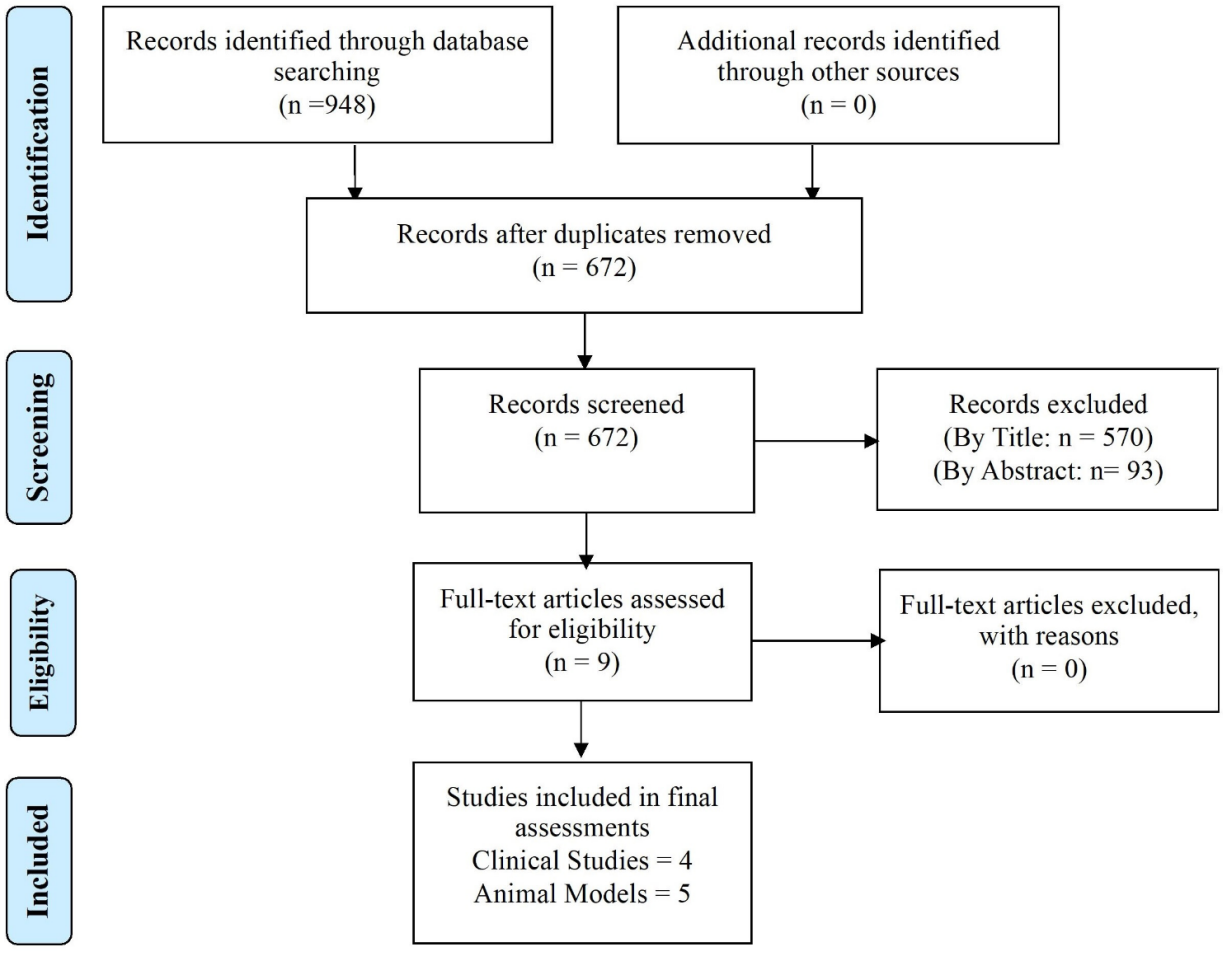
Study flow diagram

Figure 1 illustrates the systematic review’s flow diagram. In the initial search, 948 articles were potentially relevant (236 from PubMed, 296 from Web of Science, and 416 from Scopus). After removing duplicates, 672 studies remained. A preliminary screening of titles and abstracts excluded 663 records that didn’t meet inclusion criteria. The eligibility of the remaining 9 full texts was assessed, and all meeting the criteria were included in the final assessments.

Table 1 summarizes the main characteristics of the clinical studies included. These four trials were carried out from 2022 to 2023. All trials were conducted in Iran. The sample size of trials ranged from 40 to 53 participants. The intervention dosage ranged from 6 mg/day to 12 mg/day and duration ranged from 6 to 12 weeks. The trials include samples from different causes of infertility including PCOS [15, 22, 23] and Endometriosis [16].

**Table 1:** Main characteristics of the included clinical studies

Study	Country	Design	Study population (gropus and Sample size in each group)	Basal characteristics	AST dosage, duration and administration route	Study outcomes	Outcome measures
Gharaei et al. (2022) [15]	Iran	Doble blinded placebo-controlled RCT	Infertile women with PCOS allocated to 1. AST(n:20) 2. Placebo (n:20)	aged 18–40	8 mg per day for 6 weeks, orally	1. Redox status 2. ART outcomes	1. Redox status: serum and FF levels of MDA, TAC, SOD, and CAT; the expression levels of Nrf2, HO-1, NQ-1, and Keap1 mRNA or proteins 2. ART outcomes: Total number of oocytes and Embryos, OMR, high-quality embryo rate, and the rates of fertilization, chemical and clinical pregnancies
Jabarpour et al. (2023) [22]	Iran	Triple blinded-placebo controlled RCT	Infertile women with PCOS allocated to 1. AST(n:27) 2. Placebo (n:26)	Aged 18–40, BMI < 35	12 mg per day for 8 weeks, orally	1. ER Stress 2. Redox status 3. ART outcomes	1. ER stress markers: The relative expression levels of GRP78 and CHOP proteins, and mRNA expression levels of CHOP, GRP78, XBP1, ATF6, and ATF4 2. Redox status: FF level of SOD, TAC, and MDA 3. ART outcome: Total number of oocytes and Embryos, OMR, high-quality embryo rate, oocytes quality (morphology), and the rates of fertilization, chemical and clinical pregnancies
Rostami et al. (2022) [16]	Iran	Triple blinded placebo-controlled RCT	Infertile women with Endometriosis allocated to 1. AST(n:25) 2. Placebo (n:25)	20 to 40 years BMI < 30	6 mg per day for 12 weeks, orally	1. Inflammation 2. Redox status 3. ART outcomes	1. Inflammation: serum and FF level of IL-1β, IL-6, and TNF-α 2. Redox status: serum and FF level of MDA, TAC, and SOD; 3. ART outcomes: Total number of oocytes, OMR, the number of high-quality embryos and transferred embryos, and the rates of fertilization, chemical, clinical, and multiple pregnancies
Jabarpour et al. (2023) [23]	Iran	Triple blinded placebo-controlled RCT	Infertile women with PCOS allocated to 1. AST(n:27) 2. Placebo (n:26)	Aged 18–40, BMI < 35	12 mg per day for 8 weeks, orally	1. Metabolic Status 2. Redox status	1. Metabolic Status: Fasting Insulin, FBS, lipid profiles (TC, LDL, HDL) and IR markers including HOMA-Ir and QUICKI 2. Redox status: Serum level of SOD, TAC, and MDA

ART: Assisted Reproductive Technology; AST: Astaxanthin; ATF: Activating Transcription Factor; BMI: Body Mass Index; CAT: Catalase; CHOP: CCAAT/Enhancer-Binding Protein Homologous Protein; ER: Endoplasmic Reticulum; FBS: Fasting Blood Glucose; FF: Follicular Fluid; GRP78: 78-kDa Glucose-Regulated Protein; HDL: High-Density Lipoprotein; HO-1: Heme Oxygenase-1; HOMA-IR: Homeostatic Model Assessment for Insulin Resistance; IL-1β: Interleukin-1 Beta; IL-6: Interleukin-6; IR: Insulin Resistance; Keap1: Kelch-like ECH-Associated Protein; LDL: low-Density Lipoprotein; MDA: Malondialdehyde; NQ-1: NADPH Quinone Oxidoreductase1; NRF2: Nuclear Factor Erythroid 2-Related Factor 2; OMR: Oocyte Maturity Rate; PCOS: Polycystic Ovary Syndrome; QUICKI: Quantitative Insulin-Sensitivity Check Index RCT: Randomized Clinical Trial; SOD: Superoxide Dismutase; TAC: Total Antioxidant Capacity; TC: Total Cholesterol; TNF-α: Tumor Necrosis Factor-Alpha; XBP1: X-Box-Binding Protein 1

Table 2 outlines the main characteristics of the included animal studies. These five studies were conducted between 2021 and 2023. Four studies were conducted in the turkey [17, 24–26] and one in Iran [18]. Four studies developed their models of reproductive disorder in Rats [17, 24–26] and one in Mice [18]. The intervention dosage ranged from 0.1 mg/kg to 100 mg/kg and the duration ranged from single dose consumption to 14 days. Some studies used oral forms and some intraperitoneal injections. There were also some studies that did not report the exact route or duration of administration. The studies include experimental models of PCOS [17, 18] and ovarian injury [24–26].

**Table 2:** Main characteristics of the included animal studies

Study	Country	Species	Breed	Animals age	Design	Model (method of model development)	Study groups (sample size in each group)	AST dosage, duration, and administration route (dissolved)	Study outcomes	Outcome measures	Signaling pathway
Ebrahimi et al. (2021) [18]	Iran	Mice	BALB C	25–30 Days	Confirmatory Experimental with Negative, Positive, and Sham Control Groups	PCOS (S/C Injection of DHEA)	Total = 48, including: 1. NC 2. PCOS Control (PC) 3. DMSO (SC) 4. AST 5. MTF 6. AST + MTF (*n* = 8 per group)	0.1 mg/kg, (DMSO); Duration was not mentioned properly.	1. Redox status 2. Apoptosis 3. AKT Signaling 4. Ovarian Tissue Histomorphology	1. Redox status: Intracellular ROS 2. Apoptosis: Annexin V 3. AKT signaling: AKT Protein Kinase Expression 4. Ovarian tissue histomorphology: cyst formation, histomorphology of follicles at different stages of development, stroma looseness, the vascular network	AKT1,2,3
**Gunyeli** et al. (2021) [24]	Turkey	Rat	Wistar	12 Months	Confirmatory Experimental with Sham and Positive Control Groups.	GUT Damage (IP Injection of MTX)	Total = 24, including: 1. NC 2. MTX (PC) 3. AST (*n* = 8 per group)	100 mg/kg, 7 Days, Oral (Probably Saline)	1. Redox status 2. Inflammation 3. Apoptosis 4. GUT Histomorphology	1. Redox status in whole GUT (including ovaries, fallopian tube, uterus, cervix, vagina, and urethra): TAS, TOS, OSI 2. Inflammation: CRP, iNOS, G-CSF (IHC) 3. Apoptosis: caspase-3 (IHC) 4. Details were not mentioned. Only hyperemia was reported in results section	None
**Kukurt** et al. (2022) [26]	Turkey	Rat	Wistar	8–10 Weeks	Confirmatory Experimental with Sham and Positive Control Groups.	Ovarian Damage (IP Injection of 3NPA)	Total = 32, including: 1. PBS (SC) 2. AST 3. 3-NPA (PC) 4. 3-NPA + AST (*n* = 8 per group)	80 mg/kg, 14 Days, IP (PBS)	1. Redox status 2. Lipid Profile 3. Ovarian Tissue Histomorphology	1. Redox status: Plasma and ovarian tissue TAC, TOC, and OSI levels, whole blood GSH, plasma PON1 activity, MDA, NO, TSA, and TT concentrations 2. Lipid profile: HDL, TC, LDL, and TG 3. Ovarian histomorphology: histomorphology of follicles and oocytes at different stages of development, organization GCs, Presence of necrotic changes	None
**Toktay** et al. (2022) [25]	Turkey	Rat	Sprague-Dawley	10–12 Weeks	Confirmatory Experimental with Sham and Positive Control Groups.	IRI (Vascular Clamps)	Total = 42, including: 1. NC, 2. I (PC) 3. I + AST50 4. I + AST100 5. I/R (PC) 6. I/R + AST50 7. I/R + AST100 (*n* = 6 per group)	Two doses of 50 & 100 mg/kg, single dose 1 h before model induction, Oral (distilled water)	1. Redox status 2. Apoptosis 3. Inflammation 4. ovarian Tissue Histomorphology	1. Redox status: SOD activity and MDA level 2. Apoptosis: Caspase 3 expression 3. Inflammation: IL-1 β, and IL-6 expression 4. Ovarian tissue histomorphology: hemorrhagic and edematous areas, presence of apoptotic and necrotic cells, and accumulation of inflammatory cells	None
**Toktay**,** E** 2023 [17]	Turkey	Rat	Sprague-Dawley	10–12 Weeks	Confirmatory Experimental with Negative and Positive Control Groups.	PCOS (Oral Consumption of Letrozole)	Total = 72, including: 1.NC 2. PCOS (PC) 3. PCOS + MET 4. PCOS + AST10 5. PCOS + AST20 6. PCOS + AST40 7. PCOS + MET + AST10 8. PCOS + MET + AST20 9. PCOS + MET + AST40 (*n* = 8 per group)	AST 10, 20 and 40 mg/kg Doses for 7 Days; (Administration route and dissolvent were not mentioned.)	1. Redox status 2. Inflammation 3. Ovarian Tissue Histomorphology	1. Redox status: SOD activity and MDA level 2. Inflammation: TNF-α, NF-κB and IL-6 expression levels (IHC) 3. Ovarian tissue histomorphology: cyst formation, presence of normal follicles at different stages of development, apoptotic and necrotic cells	NF-κB

3NPA: 3-Nitropropionic Acid; AKT: Protein Kinase B; AST: Astaxanthin; CAT: Catalase; CRP: C-Reactive Protein; DHEA: Dehydroepiandrosterone; DMSO: Dimethyl Sulfoxide; G-CSF: Granulocyte Colony-Stimulating Factor; GSH: Glutathione; GUT: Genitourinary Tissue; HDL: High-density Lipoprotein; IHC: Immunohistochemical; IL-1β: Interleukin-1 Beta; IL-6: Interleukin-6; iNOS: Inducible Nitric Oxide Synthase; IP: Intraperitoneal; IRI: Ischemia/Reperfusion Injury; LDL: Low-density Lipoprotein; MDA: Malondialdehyde; MTF: Metformin; MTT: 3-(4,5-Dimethylthiazol-2-Yl)-2,5-Diphenyltetrazolium Bromide; MTX: Methotrexate; NC: Negative Control; NF-κB: Nuclear Factor Kappa B, NO: Nitric Oxide; OSI: Oxidative Stress Index; PBS: Phosphate-Buffered Saline; PC: Positive Control; PCOS: Polycystic Ovary Syndrome; PON1: Paraoxonase 1; ROS: Reactive Oxygen Species; S/C: Subcutaneous; SC: Sham Control; SOD: Superoxide Dismutase; TAC: Total Antioxidant Capacity; TAS: Total Antioxidant Status; TC: Total Cholesterol; TG: Triglyceride; TNF-α: Tumor Necrosis Factor-alpha; TOC: Total Oxidant Capacity; TOS: Total Oxidant Status; TSA: Total Sialic Acid; TT: Total Thiol

Through a hand search of grey literature, a total of nine clinical trials aiming at the evaluation of the effect of AST on reproductive performance, fertility, or ART outcomes were retrieved, of which 4 articles have not yet been published (Supplementary file S3).

### Risk of bias assessment

#### Clinical studies

**Fig. 2:**
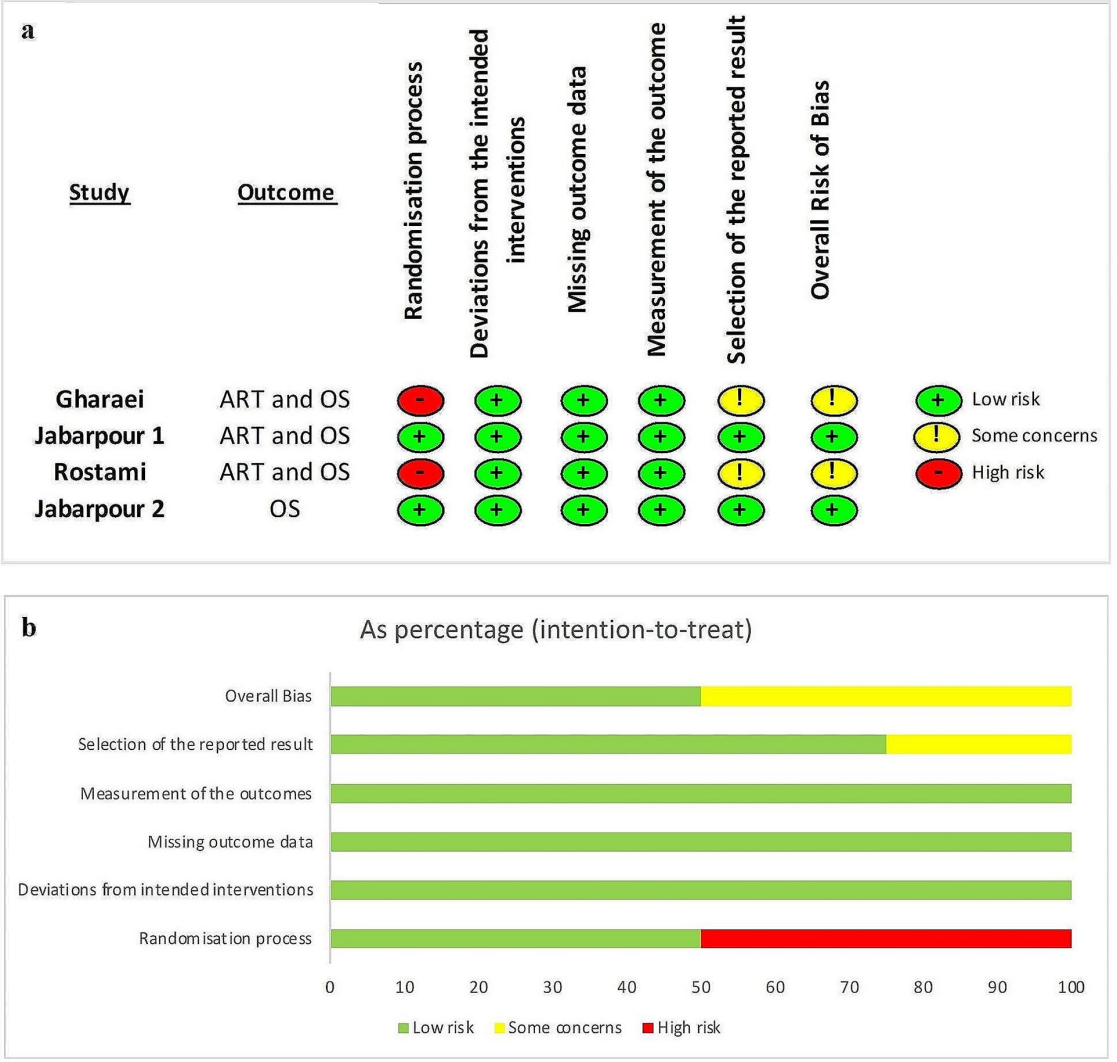
Risk of bias in clinical trials according to ROB 2 Toll; (**a**) Methodological risk of bias summary: review authors’ judgments about each methodological bias item for each included study; (**b**) Methodological risk of bias graph: review authors’ judgments about each methodological bias item presented as percentages across all included studies; OS: oxidative stress, ART: assisted reproductive technology

The summary of the risk of bias assessments for clinical studies according to the review authors’ judgements about each methodological bias is shown in Fig. 2. The detailed risk of bias assessments with review author’s comments for each individual clinical study is presented in supplementary file S4.

All four studies were randomized and reported their sequence generation methods, so they had low risk in this domain, while selection bias was at high risk in two studies [15, 16] because they did not report concealment. All four studies had a low risk of bias for performance, detection, and attrition domains. We obtained the study protocols for all four studies, but we found some reporting bias in two studies [15, 16]. These studies had some inconsistencies between their protocol and their reported outcomes, and some of their statistical analyses seemed to be post hoc.

#### Animal models

The summary of the risk of bias assessments for animal studies are shown in Table 3. Also, details of the risk of bias assessments with the reviewer’s comments for each individual animal model study are presented in supplementary file S5.

We evaluated the risk of bias for each animal study using the SYRCLE Risk of Bias Assessment Tool. We found that all five studies had high risk of bias in several domains, such as allocation concealment, random housing, blinding, and random outcome assessment. Only one study [26] had high risk of bias in sequence generation, while the others had unclear risk. All studies had low risk of bias in baseline characteristics and most of them (except for [24]) had low risk of bias in selective outcome reporting. One study had high risk of bias in incomplete outcome data [24], while one study [18] had unclear risk. The rest had low risk of bias in this domain.

**Table 3:** Summary of potential sources of bias in animal model studies according to SYRCLE risk of bias assessment tool

Author, Year	Selection bias			Performance bias		Detection bias		Attrition bias	
	Sequence generation	Baseline characteristics	Allocation concealment	Random housing	Blinding	Random outcome assessment	Blinding	Incomplete outcome data	Selective outcome reporting
Ebrahimi, 2021	unclear	low risk	high risk	high risk	high risk	high risk	high risk	unclear	low risk
Gunyeli, 2021	unclear	low risk	high risk	high risk	high risk	high risk	unclear	high risk	high risk
Kukurt, 2022	high risk	low risk	high risk	high risk	high risk	high risk	high risk	low risk	low risk
Toktay, 2022	unclear	low risk	high risk	high risk	high risk	high risk	high risk	low risk	low risk
Toktay, 2023	unclear	low risk	high risk	high risk	high risk	high risk	high risk	low risk	low risk

### Meta-analysis of clinical studies

#### Effect of AST on ART outcomes

**Fig. 3:**
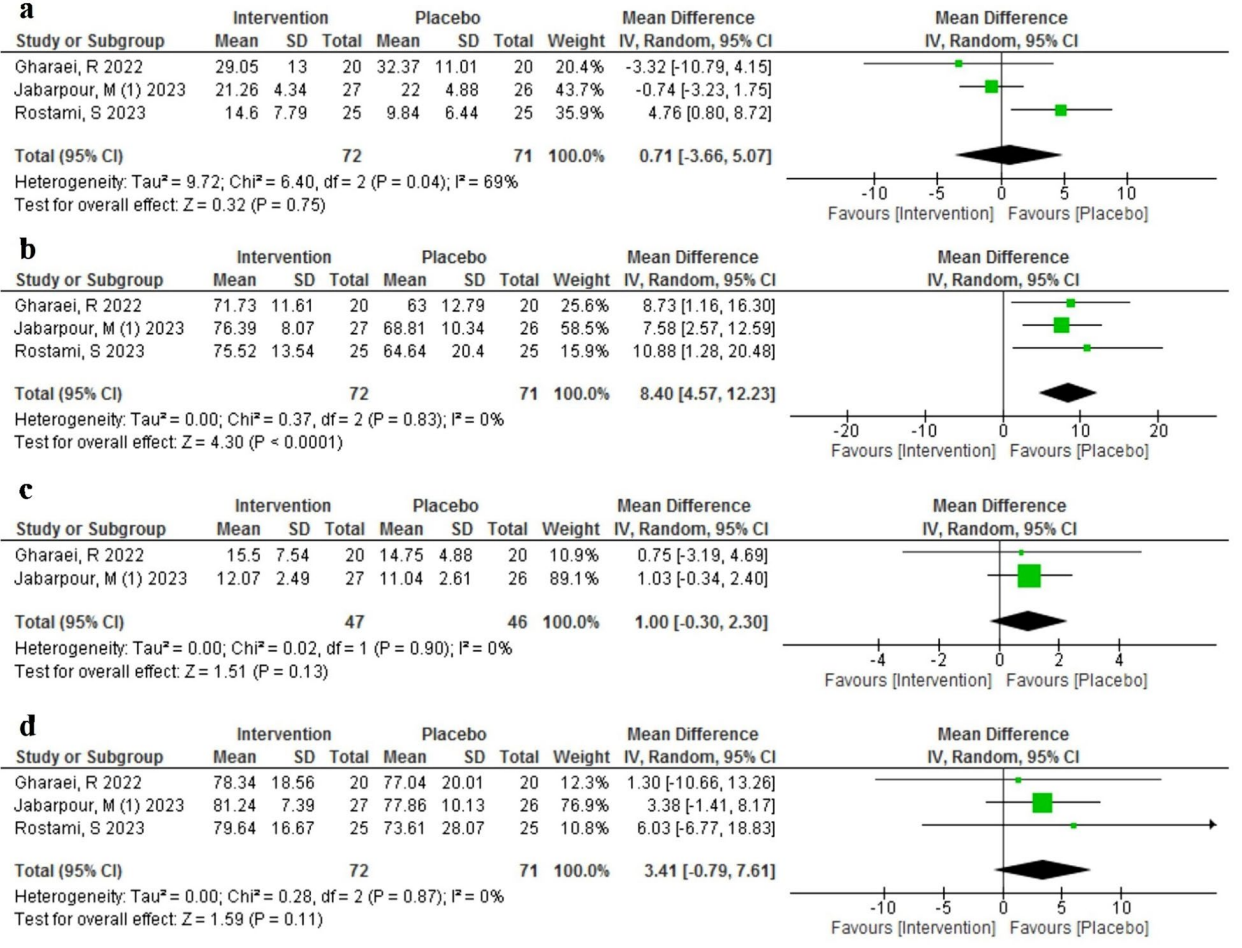
Meta-analysis of mean difference of the effect of Astaxanthin (intervention) compared to the placebo in different ART outcomes; (**a**) total number of retrieved oocytes, (**b**) oocytes maturity rate, (**c**) total number of embryos, (**d**) fertilization rate

We assessed the effect of AST (*n* = 72) compared to placebo (*n* = 71) on four ART outcomes, including the total number of retrieved oocytes, OMR, total number of embryos, and fertilization rate in three studies [15, 16, 22] with a total of 143 infertile participants. The total number of embryos was not reported in Rostami et al. [16]. The results are summarized in Fig. 3. There was no significant difference between the AST and placebo groups in the total number of retrieved oocytes (MD = 0.71, 95% CI: -3.66 to 5.07, *p* = 0.75). However, there was a high heterogeneity among the three studies included in this analysis (I^2^ = 69%).

The OMR was significantly higher in the AST group than in the placebo group (MD = 8.40, 95% CI: 4.57 to 12.23, *p* < 0.0001), with no heterogeneity among the three studies (I^2^ = 0%).

The total number of embryos and the fertilization rate were also higher in the AST group than in the placebo group, but the differences were not statistically significant (MD = 1.00, 95% CI: -0.30 to 2.30, *p* = 0.13 for total number of embryos; MD = 3.41, 95% CI: -0.79 to 7.61, *p* = 0.11 for fertilization rate). There was no heterogeneity among the two studies included in these analyses (I^2^ = 0% for both outcomes).

#### Effect of AST on pregnancy outcomes

We assessed the effect of AST compared to placebo on chemical and clinical pregnancy rates. The rates of miscarriage and live birth were reported in none of the included studies.

**Fig. 4:**
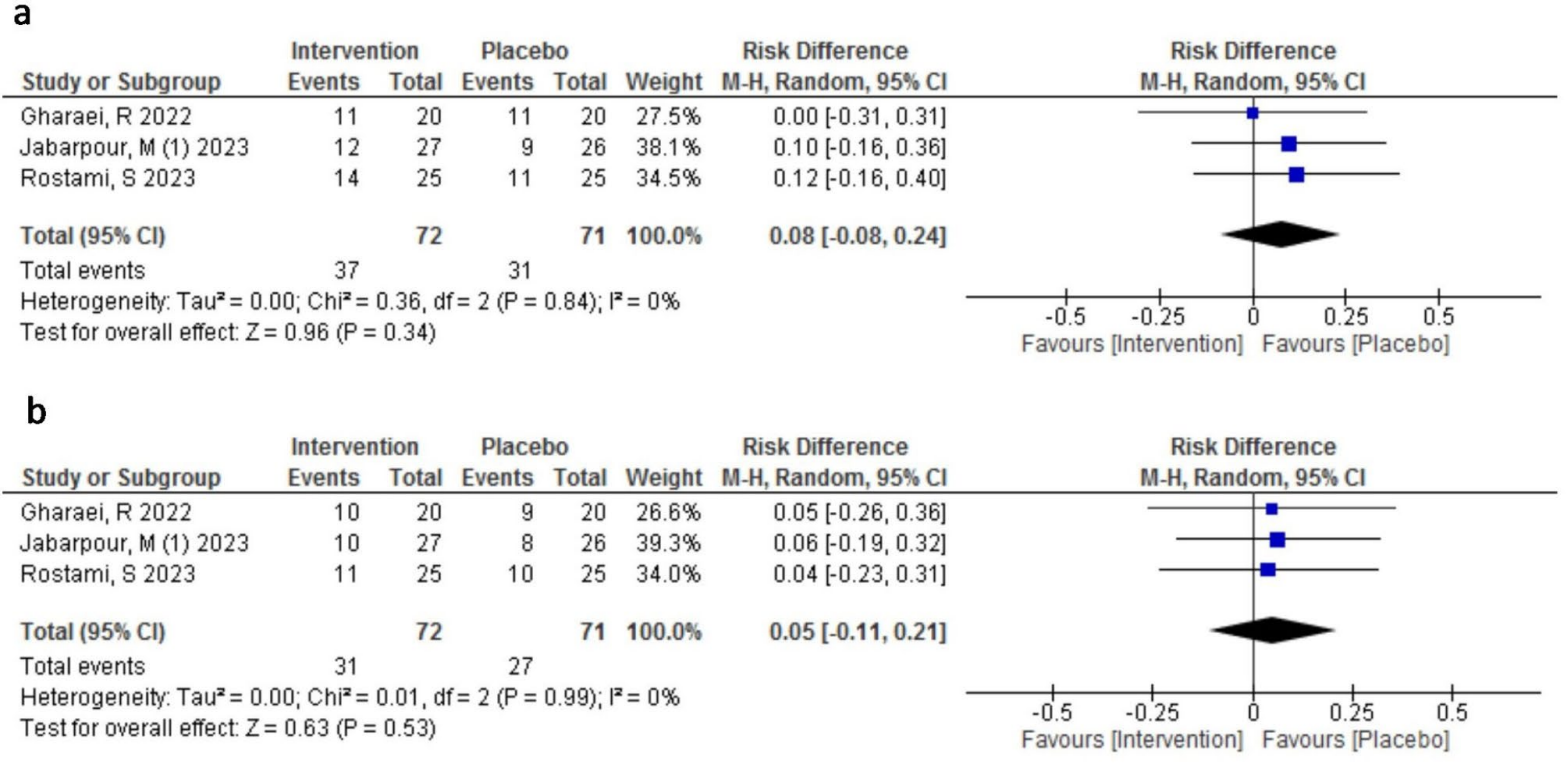
Meta-analysis of risk difference of the effect of Astaxanthin (intervention) compared to the placebo on the chemical pregnancy and clinical pregnancy; (**a**) the chemical pregnancy (**b**) the clinical pregnancy

There were three studies [15, 16, 22] enrolling 143 infertile women underwent ART that compared pregnancy outcomes between AST (n:72) and placebo group (n:71). The results are shown in Fig. 4 and supplementary file S6. The mean rate of chemical pregnancy was higher in the AST group than in the placebo group (51% vs. 44%), but the difference was not statistically significant (RR = 1.17, 95% CI: 0.83 to 1.64, *p* = 0.38). In consonance, the risk difference (RD) was 8% in favor of the AST group compared with the placebo group (RD: 0.08, 95% CI: -0.08 to 0.24; *P* = 0.34). There was no heterogeneity among the three studies included in this analysis (I^2^ = 0%).

The clinical pregnancy rate was also higher in the AST group than in the placebo group (43% vs. 38%), but the difference was not statistically significant (RR = 1.24, 95% CI: 0.63 to 2.43, *p* = 0.53). In consonance, the risk difference (RD) was only 5% in favor of the AST group compared with the placebo group (RD: 0.05, 95% CI: -0.11 to 0.21; *P* = 0.53). There was no heterogeneity among the three studies included in this analysis (I^2^ = 0%).

#### Antioxidative, anti-inflammatory and antiapoptotic effect of AST in clinical studies

In our meta-analysis of clinical studies, redox status markers were consistently reported, allowing for a robust quantitative synthesis. All included clinical studies provided data on these markers, which were subjected to meta-analysis. In contrast, inflammatory cytokines were reported in a single study [16], precluding a meta-analysis for this marker due to insufficient data. Similarly, apoptosis markers were not reported in any of the included clinical studies, highlighting a gap in the current literature and an opportunity for future research.

#### Effect of AST on redox status markers in FF

**Fig. 5:**
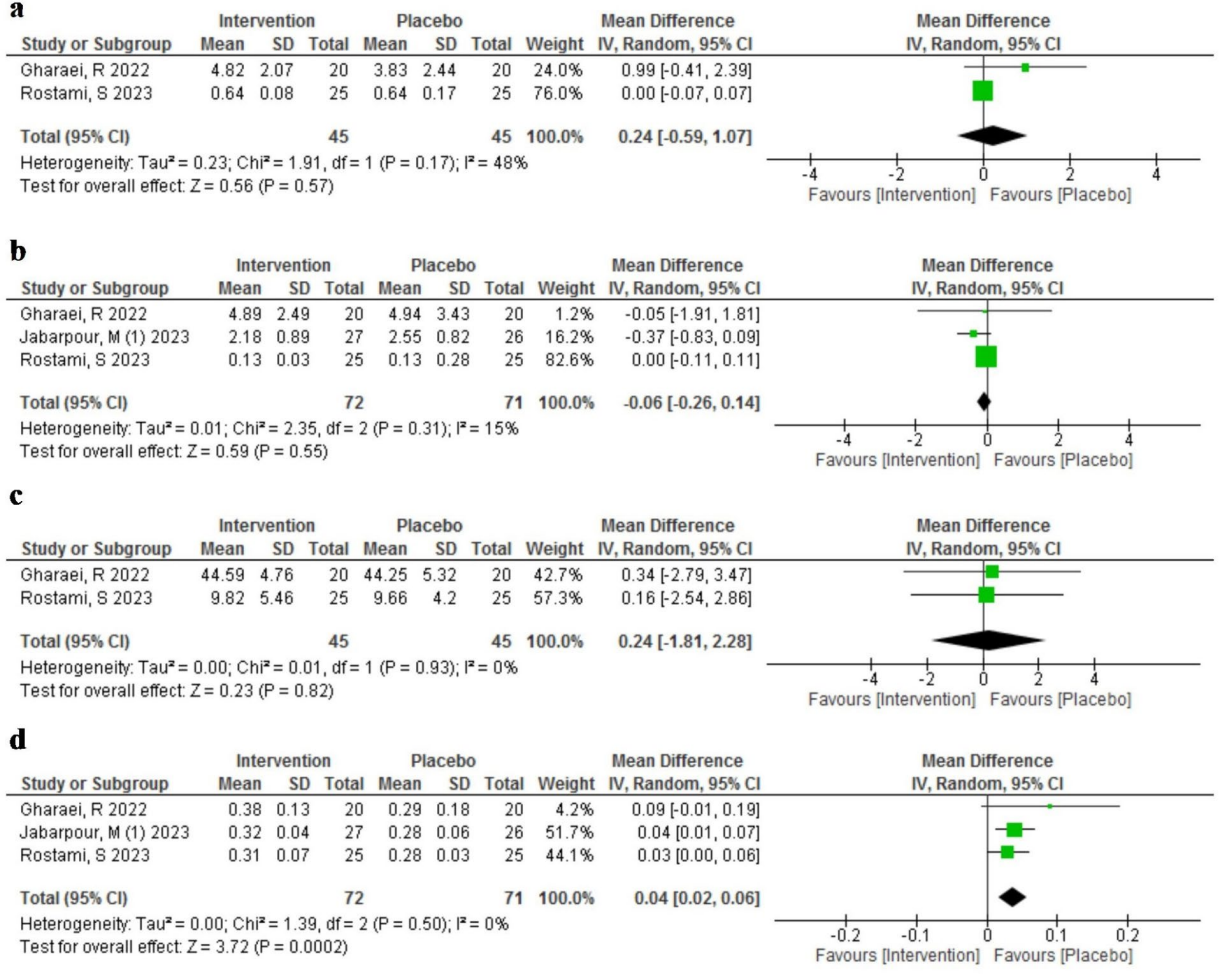
Meta-analysis of mean difference of the effect of Astaxanthin (intervention) compared to the placebo on different Redox status markers in Follicular fluid; (**a**) Catalase, (**b**) Malondialdehyde, (**c**) Superoxide dismutase, (**d**) Total Antioxidant Capacity

We compared the level of redox status markers between AST (*n* = 72) and placebo group (*n* = 71) in three studies [15, 16, 22] with a total of 143 infertile participants. The results are summarized in Fig. 5.

We measured four markers of redox status: MDA, TAC, SOD, and CAT. All three studies reported MDA, TAC, and SOD levels, while CAT level was reported only by Rosatmi et al. [16] and Gharaei et al. [15]. Gharaei et al. and Rostami et al. used the same enzyme-linked immunosorbent assay (ELISA) (ZellBio GmbH, Germany) that indirectly measured SOD activity by the reduction of a water-soluble tetrazolium salt by superoxide anion at 450 nm. Jabarpour et al. [22] used a different ELISA kit (NAVAND, Iran) that directly measured SOD activity by the inhibition of pyrogallol autoxidation by superoxide anion at 420 nm. These two methods had different sensitivity, specificity, and assay ranges for SOD measurement, which may explain the outlier value reported by Jabarpour et al. Therefore, we excluded Jabarpour et al. from our meta-analysis, as their data were not comparable with the other studies.

There was no significant difference between the AST and placebo groups in the pooled mean difference of CAT (MD = 0.24, 95% CI [-0.59, 1.07], *p* = 0.57) or MDA (MD = -0.06, 95% CI [-0.26, 0.14], *p* = 0.55) in FF. There was also no significant difference between the groups in the pooled mean difference of SOD (MD = 0.24, 95% CI [-1.81, 2.28], *p* = 0.82) in FF. However, the pooled mean difference of TAC was significantly higher in the AST group than in the placebo group (MD = 0.04, 95% CI [0.02, 0.06], *p* = 0.0002) in FF. There was no heterogeneity among the studies for any of the outcomes.

#### Effect of AST on redox status markers in serum

**Fig. 6:**
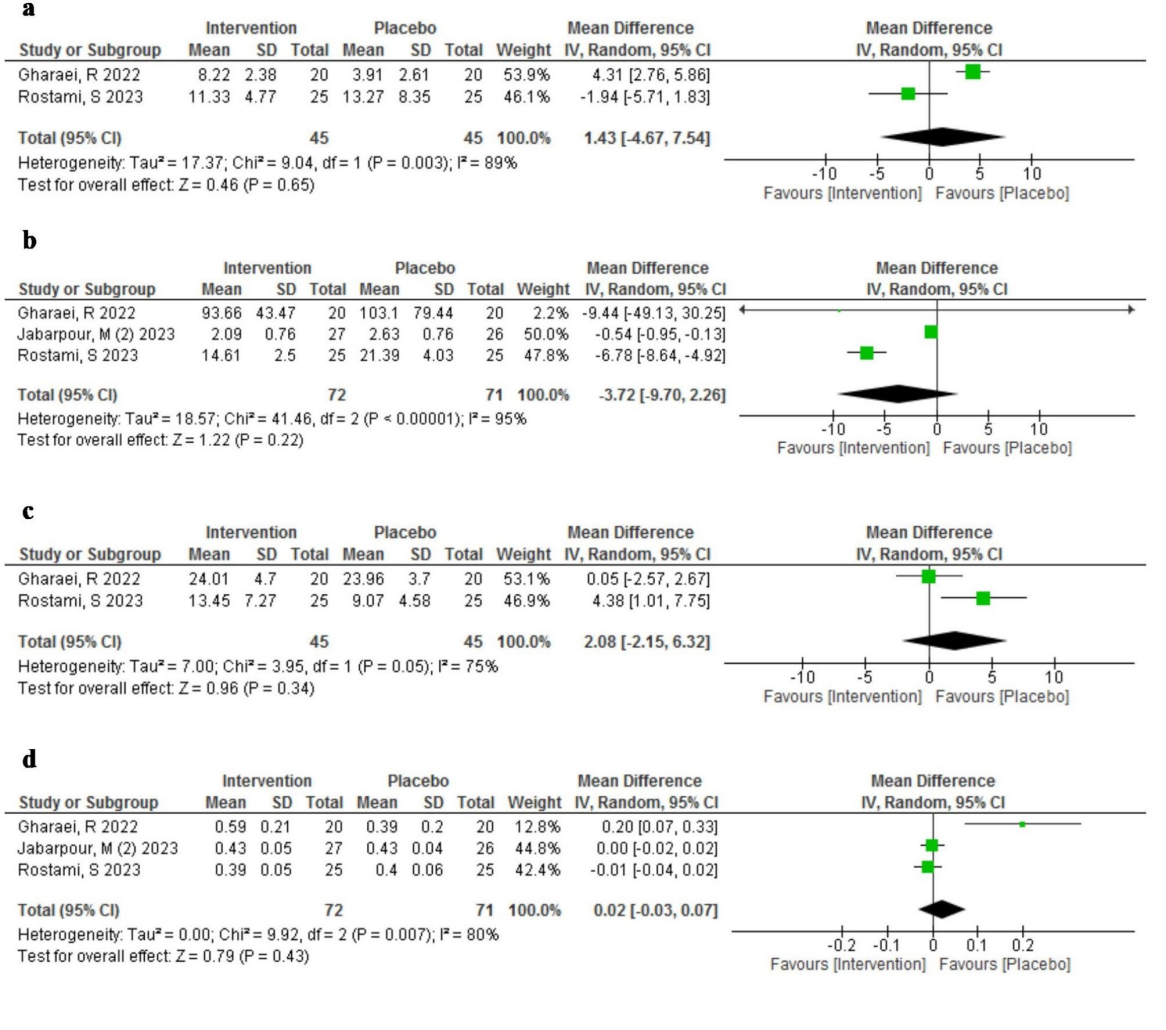
Meta-analysis of mean difference of the effect of Astaxanthin (intervention) compared to the placebo on different Redox status markers in serum; (**a**) Catalase, (**b**) Malondialdehyde, (**c**) Superoxide dismutase, (**d**) Total Antioxidant Capacity

We compared the level of redox status markers of serum between AST (*n* = 72) and placebo group (*n* = 71) in three studies [15, 16, 23] with a total of 143 infertile participants. The results are summarized in Fig. 6.

We measured four markers of redox status: MDA, TAC, SOD, and CAT. All three studies reported MDA, TAC, and SOD levels, while CAT level was reported only by Rosatmi et al. [16] and Gharaei et al. [15]. Also, like the redox status markers of FF, the SOD measurement methods were different, so just the Gharaei et al. and Rostami et al. that used the same ELISA kit (ZellBio GmbH, Germany) were included in meta-analysis.

As shown in Fig. 6a, the pooled mean difference of CAT was higher in the serum of AST group than in the placebo group, but not significantly (MD = 1.43, 95% CI: -4.67 to 7.54). As shown in Fig. 6b, the pooled mean difference of MDA in serum was lower in the AST group than in the placebo group, but not significantly (MD = -3.72, 95% CI: -9.70 to 2.26). As shown in Fig. 6c, the pooled mean difference of SOD of serum was higher in the AST group than in the placebo group, but not significantly (MD = 2.08, 95% CI: -2.15 to 6.32). As shown in Fig. 6d, the pooled mean difference of TAC of serum was also not significantly different between the groups (MD = 0.02, 95% CI: -0.03 to 0.07). There was high heterogeneity among the studies for all redox status markers of serum.

### Publication bias in clinical studies

Regarding the assessment of publication bias, our results do not include funnel plot analysis or Egger’s regression test. This decision was based on the number of studies included in our meta-analyses, which did not meet the minimum recommended threshold of 10 studies for these tests to be effective. As a result, we have not reported on publication bias, in line with best practice recommendations for systematic reviews with a limited number of studies [27].

### Descriptive synthesis of outcomes reported by animal models

The findings of the studies were heterogeneous in terms of the animal models, the doses and durations of AST treatment, the outcomes measured, and the methods used. However, some common themes emerged from the synthesis of the findings. We grouped the findings into four categories: redox status, inflammation, apoptosis, and ovarian tissue histomorphology. We summarized the findings of each individual study in Table 4.

**Table 4:** Main findings of the included animal studies

Fisrt Author	Year	Model	AST Dosage and Duration	Summary of Final Results
Ebrahimi, F	2021	PCOS	0.1 mg/kg in Combination with MTF or Alone	**(1) Redox status**: ↓ intracellular ROS in GCs; **(2) AKT signaling**: ↑ total AKT 1,2,3. **(3) Apoptosis**: An NS ↓ of apoptosis (Annexin) in the AST group; **(4) ovarian tissue histomorphology**: AST, MTF, and AST-MTF treatments improved ovarian morphology. ↑ corpus luteum and graafian follicles, ↓ primordial follicles and the emergence of primary and preantral follicles in treated groups.
Gunyeli, I	2021	GUT Damage	100 mg/kg for 7 Days	**(1) Redox status**: ↑ TAS & ↓ OSI in Ovarian tissue, and NS TAS, TOS, OSI in fallopian tubes & uterine tissue; **(2) Inflamation**: ↓ iNOS, CRP and G-CSF in whol GUT; **(3) Apoptosis**: ↓ Cas-3 in whol GUT; **(4) GUT histomorphology**: AST effectively reversed all histopathological findings, notably improving the hyperemia induced by MTX.
Kukurt, A	2022	Ovarian Damage	80 mg/kg for 14 Days	**(1) Redox status**: ↑ TAC, GSH, PON1 activity, ↓ TOC, OSI, MDA, NO, TSA, TT; **(2) lipid profile**: ↑ HDL, ↓ cholesterol, LDL and TG levels; **(3) ovarian tissue histomorphology**: loss of oocyte, disorganization in GCs and necrotic changes, especially in antral follicles was found in the 3-NPA group, while histipathological findings in in the 3‐NPA + AST group was similar to AST and Control groups.
Toktay, E	2022	Ovarian Damage	Single Doses of 50 & 100 mg/kg	**(1) Redox Status**: ↓ MDA & ↑SOD and ↑SOD in I/R + AST50 and I/R + AST100 groups; **(2) Apoptosis**: CASPASE 3 antigen in I + AST50 group was moderately and in I + AST100 and I/R + AST50 groups were mildly immune positive, while in I/R + AST100 and NC groups were immune negative; **(3) Inflamation**: Despite severly increased expression of IL-1 β, and IL-6 in PC groups immunoreactivity were decreased in both AST treatment groups (I/R + AST50 and I/R + AST100); **(4) ovarian tissue histomorphology**: In the treatment groups, especially in the I/R + AST 100, visible minimal hemorrhage areas were observed. Inflammatory and necrotic cells were not observed neither in I/R + AST50 nor I/R + AST 100 groups.
Toktay, E	2023	PCOS	10, 20 and 40 mg/kg in Combination with MTF or Alone for 7 Days	**(1) Redox status**: ↓ MDA & ↑ SOD across All AST groups in a dose-dependent manner; **(2) Inflamation**: Despite a severe increase in TNF-α, IL-6, and NF-kβ expression in PC groups, immunoreactivity decreased across all AST treatment groups particularly in combination of MTF with AST40 mg/kg; **(3) ovarian tissue histomorphology**: In PCOS group many cystic follicles, apoptotic and necrotic cells were found. In all 3 PCOS groups wich recieved AST many cortical cystic follicles were observed, while necrotic cells were just found AST 10 group; In AST20 group GCs of the secondary and Graafian follicles and in AST40 group all follicles were normal. In all 3 PCOS groups that recieved MET + AST no cortical cystic follicle was observed and many normal-looking cortical follicles were found. In both MTF + AST 20 and 40 groups no necrotic and apoptotic cells were observed

AKT: Protein Kinase B; AST: Astaxanthin; CAT: Catalase; CRP: C-Reactive Protein; G-CSF: Granulocyte Colony-Stimulating Factor; GSH: Glutathione; GUT: Genitourinary Tissue; HDL: High-density Lipoprotein; IL-1β: Interleukin-1 Beta; IL-6: Interleukin-6; iNOS: Inducible Nitric Oxide Synthase; LDL: Low-density Lipoprotein; MDA: Malondialdehyde; MTF: Metformin; MTX: Methotrexate; NC: Negative Control; NF-κB: Nuclear Factor Kappa B, NO: Nitric Oxide; OSI: Oxidative Stress Index; PC: Positive Control; PCOS: Polycystic Ovary Syndrome; PON1: Paraoxonase 1; ROS: Reactive Oxygen Species; SOD: Superoxide Dismutase; TAC: Total Antioxidant Capacity; TAS: Total Antioxidant Status; TC: Total Cholesterol; TG: Triglyceride; TNF-α: Tumor Necrosis Factor-alpha; TOC: Total Oxidant Capacity; TOS: Total Oxidant Status; TSA: Total Sialic Acid; TT: Total Thiol

#### Redox status

All studies reported that AST improved the redox status of the ovarian tissue and/or other reproductive organs by reducing OS and increasing antioxidant capacity. The indicators of redox status varied across studies, but they included markers such as ROS, MDA, TAC, total oxidant capacity, oxidative stress index, SOD, GSH, paraoxonase 1 activity, nitric oxide, thiol, and total thiol. Also, a study reported that AST improved the lipid profile by increasing high-density lipoprotein and decreasing cholesterol, low-density lipoprotein, and triglyceride levels.

#### Inflammation

Four studies reported that AST reduced the inflammation in the reproductive organs by decreasing the expression or levels of inflammatory markers, such as inducible nitric oxide synthase, C-reactive protein), granulocyte colony-stimulating factor, interleukin-1 beta, interleukin-6, tumor necrosis factor-alpha, and NF-kβ. Some studies also reported that AST improved hyperemia (increased blood flow), inflammatory cells, and necrotic changes induced by methotrexate or ischemia/reperfusion injury [24–26]. So, it seems that AST can ameliorate ovarian damage caused by inflammatory reactions.

#### Apoptosis

Three studies reported that AST reduced the apoptosis in the reproductive organs by decreasing the expression or levels of apoptotic markers, such as caspase-3 and annexin V/propidium iodide. One study also reported that AST improved the expression or levels of the anti-apoptotic signaling pathway, AKT 1,2,3 [18].

#### Ovarian tissue histomorphology

All studies reported that AST improved ovarian tissue histomorphology by increasing the number or quality of follicles, especially the graafian and corpus luteum follicles, and decreasing the number of cystic, atretic, or necrotic follicles. Some studies also reported that AST improved the GCs organization and reduced the loss of oocytes.

## Discussion

To the best of our knowledge, there hasn’t been a systematic review and meta-analysis assessing the effect of AST supplementation on female fertility and reproduction. The current review contained 4 RCTs and 5 animal studies. We found that the quality of the clinical studies was moderate to high, as two studies had a high risk of selection and reporting bias. Results of the meta-analysis of RCTs revealed that AST significantly increased the OMR and TAC in FF compared with placebo or no treatment. There was no significant difference in the other outcomes, such as the total number of oocytes, embryos, and fertilization rate, or the chemical and clinical pregnancy rates, between the groups. However, there was a high heterogeneity among the studies for the number of oocytes. We also found that AST did not significantly affect the serum levels of any of the redox status markers. Considering the high heterogeneity among the studies for all serum redox markers, these results should be interpreted with caution. The results suggested that AST had beneficial effects on ovarian function and quality and maturation of oocytes, probably by improving the local redox status in FF rather than the systemic redox status. However, considering the small number of clinical studies included and the high heterogeneity among them, these results are not precise.

The animal studies we included suggested that AST had beneficial effects on fertility and reproductive performance in subfertile or infertile models by modulating the redox status, inflammation, apoptosis, and ovarian tissue histomorphology. These studies provided evidence that AST exerts a multifaceted beneficial impact on female reproductive system performance However it should be noted that the intrinsic limitations of animal models, including species diversity and difficulty replicating human physiology, pose challenges. Systematic reviews of animal models contribute to refining methodologies but require cautious interpretation due to potential translational hurdles.

Our systematic review and meta-analysis synthesized evidence from studies that, their methodological flaws and limited sample sizes, offered valuable insights into AST’s mechanisms of action on improving fertility-related outcomes. The integration of results from both animal models and clinical trials has uncovered AST’s fundamental roles in enhancing antioxidant defense, modulating inflammation, and preventing apoptosis. These processes are crucial, as cellular impairment in ovarian cells, including GCs, theca cells, and oocytes, due to OS, inflammatory states, and apoptotic events, is known to contribute to female infertility and the pathogenesis of reproductive disorders such as PCOS, endometriosis, premature ovarian insufficiency, and poor ovarian response [28–34].

In narrowing our focus to the studies included in our review, the antioxidative actions of AST is the foremost documented mechanisms by which it exerts its beneficial effects on reproductive disorders. As observed in the study by Gharaei et al., [15] AST demonstrated the ability to enhance Nrf2 expression, improving oocyte maturation and embryo quality. AST’s unique chemical structure not only affects the Nrf2 pathway but also possesses natural protective properties against oxidative damage. It effectively neutralizes free radicals, scavenges singlet oxygen, and inhibits lipid peroxidation, safeguarding cellular membranes from oxidative damage [14].

The other AST’s mechanism of action that were proposed in included studies was its anti-inflammatory activity in animal models of ovarian damage and also endometriosis-associated infertility. The accumulation of iron in the peritoneal fluid and endometriotic cells elevates OS, which activates the NF-kB pathway in endometriotic lesions and peritoneal macrophages, contributing to the inflammatory response seen in endometriosis [35]. Activation of the NF-kB pathway increases pro-inflammatory cytokine levels, negatively impacting oocyte quality, fertilization, and embryo implantation [36]. AST prevents the degradation of inhibitor of kappa B alpha, an inhibitor of NF-κB, thereby downregulates the production of pro-inflammatory cytokines [37]. This function has potential implications for treatment of endometriosis as also showed in Rostami et al. study [16]. Furthermore, in the study conducted by Toktay et al., there was a significant increase in NF-κB expression in GCs of a PCOS rat model, providing additional insights into the potential effects of AST on these pathways [17]. It is worth noting that recent studies suggest AST’s significant impact on the complex interplay/crosstalk between Nrf2 and NF-κB pathways, where Nrf2 activation antagonizes NF-κB, potentially leading to anti-inflammatory responses [38].

Beyond antioxidative and anti-inflammatory actions of AST, its anti-apoptotic actions were also investigated. Excessive cell death, particularly apoptosis in GCs, can hinder fertility. AST showcases anti-apoptotic properties through multiple pathways, including the PI3K/Akt pathway [18]. Accordingly, Ebrahimi et al. identified a significant increase in AKT expression in GCs of a PCOS mouse model with AST treatment, confirming decreased intracellular ROS and an insignificant reduction in apoptosis [18].

Additionally, AST is suggested to have a protective effect against endoplasmic reticulum (ER) stress. ER stress in ovarian cells, can affects follicle development, oocyte maturation, and ovulation [39]. Moreover, ER stress has been associated with infertility and pathogenesis of different reproductive diseases like PCOS [40–43]. Jabarpour et al. concluded that AST can modify molecular pathways of ER stress in infertile PCOS women, influencing the expression of genes and proteins involved in the unfolding protein response and ER stress-induced apoptosis [22].

In conclusion, AST’s diverse mechanisms of action, targeting antioxidant defense, inflammation modulation, and apoptosis mitigation, suggest its potential as a therapeutic intervention for enhancing female fertility. However, it is essential to recognize that our understanding is not exhaustive. Future research should delve into other significant signaling pathways affected by AST in reproductive tissues, like mitochondrial function, autophagy, senescence, and ferroptosis. These areas remain underexplored and could unveil further dimensions of AST’s role in reproductive physiology.

One of the limitations of our meta-analysis was the high heterogeneity among the clinical studies for the serum redox status markers. This heterogeneity could be due to several factors, such as the different methods of measuring the redox status markers, the different causes of infertility of the participants, the different doses and durations of AST supplementation, and the different co-interventions or confounding factors. These factors may have affected the baseline and post-intervention levels of the redox status markers, as well as the response to AST. Therefore, the results of the serum redox status markers should be interpreted with caution, and more standardized and comparable studies are needed to assess the effect of AST on the systemic redox status in infertile women.

Another limitation of our review was the low number of clinical studies included, which reduced the precision and generalizability of our results. We only identified four RCTs that met our inclusion criteria, and all of them were conducted in Iran. This could be a strength, as it minimized the heterogeneity due to the ethnic or geographic differences among the participants. However, it could also be a weakness, as it limited the applicability of our results to other populations or settings. Therefore, more clinical studies from different countries and regions are needed to confirm the effect of AST on fertility outcomes and redox status in infertile women.

One of the reasons for the low number of studies on the effect of AST on fertility is the novelty of this research topic. AST is a relatively new antioxidant that has been gaining attention for its potential benefits on various health conditions, such as cardiovascular disease, diabetes, and neurodegenerative disorders [21]. However, the research on its effects on fertility and reproduction is still in its infancy, and most of the studies have been conducted in animal models or in vitro systems. The first clinical study on AST and fertility was published in 2022 by Gharaei et al., [15] followed by three other studies by Rostami et al. [16] and Jabarpour et al. in 2023 [22, 23].

A strength of our review was the low heterogeneity among the studies for the ART outcomes, except for the total number of retrieved oocytes. This indicated that the results were consistent and robust across the studies and that the effect of AST on these outcomes was not influenced by the sources of heterogeneity mentioned above. However, the high heterogeneity for the total number of retrieved oocytes could be due to the different causes of infertility of the participants, such as endometriosis or PCOS. In one study [16], endometriosis was the cause of infertility, while in two others [15, 22], PCOS was the cause of infertility. PCOS and endometriosis have different effects on the response to ovarian stimulation. PCOS patients generally tend to have a higher number of oocytes retrieved than other causes of infertility, due to their increased number of antral follicles and higher ovarian reserve. However, endometriosis patients tend to have a lower number of oocytes retrieved, due to their reduced ovarian reserve and the presence of endometrioma. Therefore, more studies with standardized and comparable methods are needed to assess the effect of AST on this outcome. Also, more RCTs with a focus on the underlying molecular mechanisms of AST’s action to solidify the clinical efficacy of AST in enhancing female fertility are recommended.

The animal studies that were included in our systematic review also encountered some challenges, including a limited number of studies, high risk of bias, considerable heterogeneity due to varied animal models, dosages, and treatment durations, and inconsistent outcome measures and methodological approaches. Furthermore, the analysis of qualitative markers in animal studies, such as histopathological examinations further limited the overall interpretation. Therefore, more rigorous and standardized animal studies are needed to confirm the effects of AST and to translate them to human settings.

## Conclusion

This systematic review and meta-analysis was primarily conducted to assess the impact of AST on reproductive performance, fertility, and assisted reproductive technology (ART) outcomes. Additionally, it aimed to elucidate the potential mechanisms of action of AST as a secondary objective in both infertile women and animal models of infertility and reproductive disorders. We included four clinical trials and five animal studies in our analysis. The results showed that AST significantly increased the OMR and TAC in FF, but did not have a significant effect on other ART outcomes, pregnancy outcomes, or redox status markers in FF or serum in infertile women. The animal studies suggested that AST improved the redox status, inflammation, apoptosis, and ovarian tissue histomorphology in various reproductive disorders. However, the quality of evidence was low to moderate, and the heterogeneity and inconsistency among the studies were high. Therefore, more well-designed and rigorous studies are needed to confirm the efficacy and safety of AST for improving reproductive health and outcomes.

### Electronic supplementary material

Below is the link to the electronic supplementary material.

Supplementary Material 1: PRISMA 2020 Checklist

Supplementary Material 2: Search strategy

Supplementary Material 3: Unpublished trials retrieved through the WHO clinical trials registration database

Supplementary Material 4: Details of Risk of bias assessment for Clinical Trials

Supplementary Material 5: Details of Risk of Bias Assessment in Animal Model Studies

Supplementary Material 6: Study Additional Plots

## Data Availability

The data collection forms, data extracted from included studies, and data used for all analyses used in the review are not publicly available, however, they will be available for reasonable reasons.

## Abbreviations

ART: Assisted Reproductive Technology
AST: Astaxanthin
CAT: Catalase
FF: Follicular Fluid
GCs: Granulosa Cells
GSH: Glutathione
MDA: Malondialdehyde
NF-κB: Nuclear Factor kappa B
Nrf2/HO-: 1nuclear factor erythroid 2-related factor 2/heme oxygenase-1
OMR: Oocyte Maturity Rate
OS: Oxidative stress
PCOS: Polycystic Ovary Syndrome
PI3K/Akt: Phosphoinositide 3-kinase/protein kinase B
ROS: Reactive Oxygen Species
ROS: Reactive Oxygen Species
SOD: Superoxide Dismutase
TAC: Total Antioxidant Capacity
