# Supplementary material for: Effect of astaxanthin supplementation on female fertility and reproductive outcomes: a systematic review and meta-analysis of clinical and animal studies
Source: J Ovarian Res. 2024 Aug 10;17:163. doi: 10.1186/s13048-024-01472-7 (PMC11316280; doi:10.1186/s13048-024-01472-7)
Supplement: Supplementary file 2 — Supplementary Material 2: Search strategy [file 13048_2024_1472_MOESM2_ESM.docx]

**Supplementary file 2 (S2) - Search strategy**

**Title: Effect of Astaxanthin Supplementation on Female Fertility and Reproductive Outcomes: A Systematic Review and Meta-analysis of Clinical and Animal Studies**

**Total number of studies identified through database searching: 948**

**Total number of studies identified through cite-to-cite checking or other sources: 0**

**Duplicate: 276**

**Deleted by Title Screening: 570**

**Deleted by Abstract Screening: 93**

**Search terms selection**

Search term selection was based on the PICO frame work of the study.

PICOs:

1. Population: For clinical settings, Women with reproductive disorders/pathologies, including infertility, polycystic ovary syndrome, endometriosis, diminished ovarian reserve, and ovarian aging, and for animal studies female mammalian models of a reproductive related disorders/pathologies, including PCOS, Endometriosis, aging, or experimental ovarian injuries (induced by ischemia-reperfusion, heat shock, or Toxic agents).
2. Intervention: Intervention and control: oral AST supplementation in any dosage or duration compared to placebo in clinical studies and oral consumption or intraperitoneal (IP) injection of AST compared to negative/positive/or sham control groups in animal studies.
3. Outcome: Primary outcomes: biological or physiological measures of the Fertility and Reproductive Performance. Secondary outcomes: AST's cellular mechanisms of action in reproductive disorders (redox status, inflammation, and apoptosis markers in ovarian tissue, follicular fluid [FF], or serum).

| **Search terms** | **Descriptors** |
| --- | --- |
| Population | Fertility OR Infertility OR Sterility OR Subfertility OR reproduction OR reproductive OR oocyte OR Ovocyte OR oogenesis OR ovary OR ovarian OR intracytoplasmic sperm injection OR **In Vitro Fertilization** OR ICSI OR IVF OR pregnancy OR implantation OR miscarriage OR abortion OR embryo OR fetus OR Endometriosis |
| Intervention | Astaxanthin OR Staxanthin |

**PubMed**

**Date of search:** 12/29/2023

**Number of localized studies:** 236

**Limits: No Limits**

|  | **Descriptors** | Results |
| --- | --- | --- |
| **#1** | **(((((((((((((((((((((Fertility) OR (Infertility)) OR (Sterility)) OR (Subfertility)) OR (reproduction)) OR (reproductive)) OR (oocyte)) OR (Ovocyte)) OR (oogenesis)) OR (ovary)) OR (ovarian)) OR (intracytoplasmic sperm injection)) OR (In Vitro Fertilization)) OR (ICSI)) OR (IVF)) OR (pregnancy)) OR (implantation)) OR (miscarriage)) OR (abortion)) OR (embryo)) OR (fetus)) OR (Endometriosis)** | **3,026,800** |
| **#2** | **(Astaxanthin) OR (Staxanthin)** | 3,479 |
| **#3** | **#1** AND **#2** | 236 |

**Web Of Science**

**Date of search:** 01/01/2024

**Number of localized studies:** 296

**Limits: no limit**

|  | **Descriptors** | Number of studies reached |
| --- | --- | --- |
| **#1** | **Fertility** (Topic) or **Infertility** (Topic) or **Sterility** (Topic) or **Subfertility** (Topic) or **reproducti*** (Topic) or **oocyte** (Topic) or **oogenesis** (Topic) or **ovar*** (Topic) or **intracytoplasmic sperm injection** (Topic) or **In Vitro Fertilization** (Topic) or **pregnancy** (Topic) or **implantation** (Topic) or **miscarriage** (Topic) or **abortion** (Topic) or **embryo** (Topic) or **fetus** (Topic) or **Endometriosis** (Topic) | 1,970,694 |
| **#2** | **Astaxanthin** (Topic) or **staxanthin** (Topic) | 6,753 |
| **#3** | #1 AND #2 | 296 |

**Scopus**

**Date of search:** 01/01/2024

**Number of localized studies:** 416

**Limits: no limit**

|  | **Descriptors** | Number of studies reached |
| --- | --- | --- |
| **#1** | ( TITLE-ABS-KEY ( fertility ) OR TITLE-ABS-KEY ( infertility ) OR TITLE-ABS-KEY ( sterility ) OR TITLE-ABS-KEY ( subfertility ) OR TITLE-ABS-KEY ( reproduction ) OR TITLE-ABS-KEY ( reproductive ) OR TITLE-ABS-KEY ( oocyte ) OR TITLE-ABS-KEY ( ovocyte ) OR TITLE-ABS-KEY ( oogenesis ) OR TITLE-ABS-KEY ( ovary ) OR TITLE-ABS-KEY ( ovarian ) OR TITLE-ABS-KEY ( intracytoplasmic AND sperm AND injection ) OR TITLE-ABS-KEY ( in AND vitro AND fertilization ) OR TITLE-ABS-KEY ( icsi ) OR TITLE-ABS-KEY ( ivf ) OR TITLE-ABS-KEY ( pregnancy ) OR TITLE-ABS-KEY ( implantation ) OR TITLE-ABS-KEY ( miscarriage ) OR TITLE-ABS-KEY ( abortion ) OR TITLE-ABS-KEY ( embryo ) OR TITLE-ABS-KEY ( fetus ) OR TITLE-ABS-KEY ( endometriosis ) ) | 3,482,377 |
| **#2** | (TITLE-ABS-KEY (astaxanthin) OR TITLE-ABS-KEY ( staxanthin ) ) | 7,797 |
| **#3** | **#1** AND **#2** | 416 |
