# Supplementary material for: Effect of astaxanthin supplementation on female fertility and reproductive outcomes: a systematic review and meta-analysis of clinical and animal studies
Source: J Ovarian Res. 2024 Aug 10;17:163. doi: 10.1186/s13048-024-01472-7 (PMC11316280; doi:10.1186/s13048-024-01472-7)
Supplement: Supplementary file 3 — Supplementary Material 3: Unpublished trials retrieved through the WHO clinical trials registration database [file 13048_2024_1472_MOESM3_ESM.docx]

**Supplementary file 3 (s3): Unpublished trials retrieved through the WHO clinical trials registration database**

| **Registration ID** | **Registration Date** | **Origin** | **Recruitment status** | **Health Condition studied** | **AST dosage used** | **Primary and**  **Secondary Outcomes** |
| --- | --- | --- | --- | --- | --- | --- |
| IRCT20231001059573N1 | 2023-10-10 | Iran (Esfahan University of Medical Sciences) | Recruiting | PCOS | 10 mg per day for 12 weeks | Serum MDA, lipid profile (TG, total cholesterol, LDL-c, HDL-c, cholesterol), insulin sensitivity (HOMA-IR), and severity of hirsutism |
| IRCT20221005056092N1 | 2023-05-01 | Iran (Tehran University of Medical Sciences) | Recruiting | PCOS | 6 mg per day for for 2 Months | Serum inflammatory cytokines,  Wnt/ß-catenin signaling pathway genes in GCs, and ART outcomes (fertilization rate and oocyte quality) |
| IRCT20230223057510N1 | 2023-03-16 | Iran (Tehran University of Medical Sciences) | Complete | POR | 12 mg of per day for 8 weeks | FF inflammatory cytokines (L-6, IL-8, VEGF), redox Status (MDA, TAC, SOD), Cell-free DNA and ART outcomes (fertilization rate, number and quality of oocytes, number and quality of embryos, chemical pregnancy rate), and antral follicle count. |
| IRCT20201029049183N2 | 2022-04-12 | Iran (Tehran University of Medical Sciences) | Complete | PCOS | 12 mg per day for 60 days | ER stress and apoptotic factor in PBMCs and plasma inflammatory factors |

ER: Endoplasmic Reticulum; FF: Follicular Fluid; GCs: Granulosa Cells; IL: Interleukin; MDA: Malondialdehyde; PBMCs: peripheral blood mononuclear cell; PCOS: Polycystic Ovary Syndrome; POR: Poor ovarian response, SOD: Superoxide dismutase; TAC: Total antioxidant capacity; TG: triglyceride; VEGF: Vascular endothelial growth factor;
