# Supplementary material for: Effect of astaxanthin supplementation on female fertility and reproductive outcomes: a systematic review and meta-analysis of clinical and animal studies
Source: J Ovarian Res. 2024 Aug 10;17:163. doi: 10.1186/s13048-024-01472-7 (PMC11316280; doi:10.1186/s13048-024-01472-7)
Supplement: Supplementary file 4 — Supplementary Material 4: Details of Risk of bias assessment for Clinical Trials [file 13048_2024_1472_MOESM4_ESM.docx]

**Supplementary File 4 (S4)**

**Details of Risk of bias assessment for Clinical Trials**

| **Unique ID** | 1 | **Study ID** | Gharaei et al. 2022 | | | | | **Assessor 1** | | **Assessor 2** | |
| --- | --- | --- | --- | --- | --- | --- | --- | --- | --- | --- | --- |
| **Ref or Label** |  | **Aim** | assignment to intervention (the 'intention-to-treat' effect) | | | | | **AM-H** | | **AS** | |
| **Outcome** | **ART and OS** |  | | | | | | **Source** | | Journal article(s) | |
| **Domain** | **Signalling question** | | | | | | | **Response** | **Comments** | | |
| **Bias arising from the randomization process** | 1.1 Was the allocation sequence random? | | | | | | | Y | The trial did not report any concealment. | | |
|  | 1.2 Was the allocation sequence concealed until participants were enrolled and assigned to interventions? | | | | | | | N |  |  |  |
|  | 1.3 Did baseline differences between intervention groups suggest a problem with the randomization process? | | | | | | | N |  | | |
|  | **Risk of bias judgement** | | | | | | | **High** |  | | |
| **Bias due to deviations from intended interventions** | 2.1.Were participants aware of their assigned intervention during the trial? | | | | | | | N |  | | |
|  | 2.2.Were carers and people delivering the interventions aware of participants' assigned intervention during the trial? | | | | | | | N |  |  |  |
|  | 2.3. If Y/PY/NI to 2.1 or 2.2: Were there deviations from the intended intervention that arose because of the experimental context? | | | | | | | NA |  | | |
|  | 2.4 If Y/PY to 2.3: Were these deviations likely to have affected the outcome? | | | | | | | NA |  | | |
|  | 2.5. If Y/PY/NI to 2.4: Were these deviations from intended intervention balanced between groups? | | | | | | | NA |  | | |
|  | 2.6 Was an appropriate analysis used to estimate the effect of assignment to intervention? | | | | | | | Y | Authors stated that "intergroup comparisons of the chemical and clinical pregnancy rates were performed using Fisher’s exact and chi-squared tests". It seems that there was a typographical error, as "between group" is correct in this case, not intergroup. | | |
|  | 2.7 If N/PN/NI to 2.6: Was there potential for a substantial impact (on the result) of the failure to analyse participants in the group to which they were randomized? | | | | | | | NA |  | | |
|  | **Risk of bias judgement** | | | | | | | **Low** |  | | |
| **Bias due to missing outcome data** | 3.1 Were data for this outcome available for all, or nearly all, participants randomized? | | | | | | | Y |  | | |
|  | 3.2 If N/PN/NI to 3.1: Is there evidence that result was not biased by missing outcome data? | | | | | | | NA |  | | |
|  | 3.3 If N/PN to 3.2: Could missingness in the outcome depend on its true value? | | | | | | | NA |  | | |
|  | 3.4 If Y/PY/NI to 3.3: Is it likely that missingness in the outcome depended on its true value? | | | | | | | NA |  |  |  |
|  | **Risk of bias judgement** | | | | | | | **Low** |  | | |
| **Bias in measurement of the outcome** | 4.1 Was the method of measuring the outcome inappropriate? | | | | | | | N |  | | |
|  | 4.2 Could measurement or ascertainment of the outcome have differed between intervention groups? | | | | | | | N |  | | |
|  | 4.3 Were outcome assessors aware of the intervention received by study participants? | | | | | | | N |  | | |
|  | 4.4 If Y/PY/NI to 4.3: Could assessment of the outcome have been influenced by knowledge of intervention received? | | | | | | | NA |  | | |
|  | 4.5 If Y/PY/NI to 4.4: Is it likely that assessment of the outcome was influenced by knowledge of intervention received? | | | | | | | NA |  |  |  |
|  | **Risk of bias judgement** | | | | | | | **Low** |  | | |
| **Bias in selection of the reported result** | 5.1 Were the data that produced this result analysed in accordance with a pre-specified analysis plan that was finalized before unblinded outcome data were available for analysis? | | | | | | | PN | The study protocol, available online at the IRCT site, listed changes in hormonal profile as one of the primary outcomes. However, the published article did not report this outcome. Moreover, the protocol did not specify ART outcomes, which the article presented as the main results. | | |
|  | 5.2 ... multiple eligible outcome measurements (e.g. scales, definitions, time points) within the outcome domain? | | | | | | | N |  | | |
|  | 5.3 ... multiple eligible analyses of the data? | | | | | | | PY | It seems that the authors may have committed outcome reporting bias by adding a correlation analysis that was not relevant to the study aims, so we judged the correlation analysis was indeed an unplanned and unjustified addition to the study results. | | |
|  | **Risk of bias judgement** | | | | | | | **Some concerns** |  | | |
| **Overall bias** | **Risk of bias judgement** | | | | | | | **Some concerns** |  | | |
|  |  |  | | | | |  |  |  | | |
|  |  |  | | | | |  |  |  | | |
| **Unique ID** | 2 | **Study ID** | | Jabarpour Jabarpour et al, 2023 (1) | | | | **Assessor 1** | | | **Assessor 2** |
| **Ref or Label** |  | **Aim** | | assignment to intervention (the 'intention-to-treat' effect) | | | | **AM-H** | | | **AS** |
| **Outcome** | ART and OS |  |  |  |  |  |  | **Source** | | | Journal article(s) |
| **Domain** | **Signalling question** | | | | | | | **Response** | **Comments** | | |
| **Bias arising from the randomization process** | 1.1 Was the allocation sequence random? | | | | | | | Y |  | | |
|  | 1.2 Was the allocation sequence concealed until participants were enrolled and assigned to interventions? | | | | | | | Y |  |  |  |
|  | 1.3 Did baseline differences between intervention groups suggest a problem with the randomization process? | | | | | | | N |  | | |
|  | **Risk of bias judgement** | | | | | | | **Low** |  | | |
| **Bias due to deviations from intended interventions** | 2.1.Were participants aware of their assigned intervention during the trial? | | | | | | | N |  | | |
|  | 2.2.Were carers and people delivering the interventions aware of participants' assigned intervention during the trial? | | | | | | | N |  |  |  |
|  | 2.3. If Y/PY/NI to 2.1 or 2.2: Were there deviations from the intended intervention that arose because of the experimental context? | | | | | | | NA |  | | |
|  | 2.4 If Y/PY to 2.3: Were these deviations likely to have affected the outcome? | | | | | | | NA |  | | |
|  | 2.5. If Y/PY/NI to 2.4: Were these deviations from intended intervention balanced between groups? | | | | | | | NA |  | | |
|  | 2.6 Was an appropriate analysis used to estimate the effect of assignment to intervention? | | | | | | | Y |  | | |
|  | 2.7 If N/PN/NI to 2.6: Was there potential for a substantial impact (on the result) of the failure to analyse participants in the group to which they were randomized? | | | | | | | NA |  | | |
|  | **Risk of bias judgement** | | | | | | | **Low** |  | | |
| **Bias due to missing outcome data** | 3.1 Were data for this outcome available for all, or nearly all, participants randomized? | | | | | | | Y |  | | |
|  | 3.2 If N/PN/NI to 3.1: Is there evidence that result was not biased by missing outcome data? | | | | | | | NA |  | | |
|  | 3.3 If N/PN to 3.2: Could missingness in the outcome depend on its true value? | | | | | | | NA |  | | |
|  | 3.4 If Y/PY/NI to 3.3: Is it likely that missingness in the outcome depended on its true value? | | | | | | | NA |  |  |  |
|  | **Risk of bias judgement** | | | | | | | **Low** |  | | |
| **Bias in measurement of the outcome** | 4.1 Was the method of measuring the outcome inappropriate? | | | | | | | N |  | | |
|  | 4.2 Could measurement or ascertainment of the outcome have differed between intervention groups? | | | | | | | N |  | | |
|  | 4.3 Were outcome assessors aware of the intervention received by study participants? | | | | | | | N |  | | |
|  | 4.4 If Y/PY/NI to 4.3: Could assessment of the outcome have been influenced by knowledge of intervention received? | | | | | | | NA |  | | |
|  | 4.5 If Y/PY/NI to 4.4: Is it likely that assessment of the outcome was influenced by knowledge of intervention received? | | | | | | | NA |  |  |  |
|  | **Risk of bias judgement** | | | | | | | **Low** |  | | |
| **Bias in selection of the reported result** | 5.1 Were the data that produced this result analysed in accordance with a pre-specified analysis plan that was finalized before unblinded outcome data were available for analysis? | | | | | | | Y |  | | |
|  | 5.2 ... multiple eligible outcome measurements (e.g. scales, definitions, time points) within the outcome domain? | | | | | | | N |  | | |
|  | 5.3 ... multiple eligible analyses of the data? | | | | | | | N |  | | |
|  | **Risk of bias judgement** | | | | | | | **Low** |  | | |
| **Overall bias** | **Risk of bias judgement** | | | | | | | **Low** |  | | |
|  |  |  | | | | |  |  |  | | |
|  |  |  | | | | |  |  |  | | |
| **Unique ID** | 3 | **Study ID** | | | Rostami et al. 2023 | | | **Assessor 1** | | | **Assessor 2** |
| **Ref or Label** |  | **Aim** | | | assignment to intervention (the 'intention-to-treat' effect) | | | **AM-H** | | | **AS** |
| **Outcome** | ART and OS |  |  |  |  |  |  | **Source** | | | Journal article(s) |
| **Domain** | **Signalling question** | | | | | | | **Response** | **Comments** | | |
| **Bias arising from the randomization process** | 1.1 Was the allocation sequence random? | | | | | | | Y | There seems to be a discrepancy netween the sample size calculation in methods section and the actual number of participants randomized in the study. Also, no concealment was reported. | | |
|  | 1.2 Was the allocation sequence concealed until participants were enrolled and assigned to interventions? | | | | | | | N |  |  |  |
|  | 1.3 Did baseline differences between intervention groups suggest a problem with the randomization process? | | | | | | | N |  | | |
|  | **Risk of bias judgement** | | | | | | | **High** |  | | |
| **Bias due to deviations from intended interventions** | 2.1.Were participants aware of their assigned intervention during the trial? | | | | | | | N |  | | |
|  | 2.2.Were carers and people delivering the interventions aware of participants' assigned intervention during the trial? | | | | | | | N |  |  |  |
|  | 2.3. If Y/PY/NI to 2.1 or 2.2: Were there deviations from the intended intervention that arose because of the experimental context? | | | | | | | NA |  | | |
|  | 2.4 If Y/PY to 2.3: Were these deviations likely to have affected the outcome? | | | | | | | NA |  | | |
|  | 2.5. If Y/PY/NI to 2.4: Were these deviations from intended intervention balanced between groups? | | | | | | | NA |  | | |
|  | 2.6 Was an appropriate analysis used to estimate the effect of assignment to intervention? | | | | | | | PY | The specific statistical test used to analyze medication effectiveness between the two groups is not explicitly mentioned in the Statistical Methods section. However, table 2, in the results section, appears to present findings that could only have been generated through such a test. | | |
|  | 2.7 If N/PN/NI to 2.6: Was there potential for a substantial impact (on the result) of the failure to analyse participants in the group to which they were randomized? | | | | | | | NA |  | | |
|  | **Risk of bias judgement** | | | | | | | **Low** |  | | |
| **Bias due to missing outcome data** | 3.1 Were data for this outcome available for all, or nearly all, participants randomized? | | | | | | | Y |  | | |
|  | 3.2 If N/PN/NI to 3.1: Is there evidence that result was not biased by missing outcome data? | | | | | | | NA |  | | |
|  | 3.3 If N/PN to 3.2: Could missingness in the outcome depend on its true value? | | | | | | | NA |  | | |
|  | 3.4 If Y/PY/NI to 3.3: Is it likely that missingness in the outcome depended on its true value? | | | | | | | NA |  |  |  |
|  | **Risk of bias judgement** | | | | | | | **Low** |  | | |
| **Bias in measurement of the outcome** | 4.1 Was the method of measuring the outcome inappropriate? | | | | | | | N |  | | |
|  | 4.2 Could measurement or ascertainment of the outcome have differed between intervention groups? | | | | | | | N |  | | |
|  | 4.3 Were outcome assessors aware of the intervention received by study participants? | | | | | | | N |  | | |
|  | 4.4 If Y/PY/NI to 4.3: Could assessment of the outcome have been influenced by knowledge of intervention received? | | | | | | | NA |  | | |
|  | 4.5 If Y/PY/NI to 4.4: Is it likely that assessment of the outcome was influenced by knowledge of intervention received? | | | | | | | NA |  |  |  |
|  | **Risk of bias judgement** | | | | | | | **Low** |  | | |
| **Bias in selection of the reported result** | 5.1 Were the data that produced this result analysed in accordance with a pre-specified analysis plan that was finalized before unblinded outcome data were available for analysis? | | | | | | | PN | The study protocol, available online at the IRCT site, did not specify Redox status markers and ART outcomes, while the article presented these outcomes in the main results. | | |
|  | 5.2 ... multiple eligible outcome measurements (e.g. scales, definitions, time points) within the outcome domain? | | | | | | | N |  | | |
|  | 5.3 ... multiple eligible analyses of the data? | | | | | | | PY | It seems that the authors may have committed outcome reporting bias by adding a correlation analysis that was not relevant to the study aims, so we judged the correlation analysis was indeed an unplanned and unjustified addition to the study results. | | |
|  | **Risk of bias judgement** | | | | | | | **Some concerns** |  | | |
| **Overall bias** | **Risk of bias judgement** | | | | | | | **Some concerns** |  | | |
|  |  |  | | | | |  |  |  | | |
|  |  |  | | | | |  |  |  | | |
| **Unique ID** | 4 | **Study ID** | | | | Jabarpour et al, 2023 (2) | | **Assessor 1** | | | **Assessor 2** |
| **Ref or Label** |  | **Aim** | | | | assignment to intervention (the 'intention-to-treat' effect) | | **AM-H** | | | **AS** |
| **Outcome** | OS |  |  |  |  |  |  | **Source** | | | Journal article(s) |
| **Domain** | **Signalling question** | | | | | | | **Response** | **Comments** | | |
| **Bias arising from the randomization process** | 1.1 Was the allocation sequence random? | | | | | | | Y |  | | |
|  | 1.2 Was the allocation sequence concealed until participants were enrolled and assigned to interventions? | | | | | | | Y |  |  |  |
|  | 1.3 Did baseline differences between intervention groups suggest a problem with the randomization process? | | | | | | | N |  | | |
|  | **Risk of bias judgement** | | | | | | | **Low** |  | | |
| **Bias due to deviations from intended interventions** | 2.1.Were participants aware of their assigned intervention during the trial? | | | | | | | N |  | | |
|  | 2.2.Were carers and people delivering the interventions aware of participants' assigned intervention during the trial? | | | | | | | N |  |  |  |
|  | 2.3. If Y/PY/NI to 2.1 or 2.2: Were there deviations from the intended intervention that arose because of the experimental context? | | | | | | | NA |  | | |
|  | 2.4 If Y/PY to 2.3: Were these deviations likely to have affected the outcome? | | | | | | | NA |  | | |
|  | 2.5. If Y/PY/NI to 2.4: Were these deviations from intended intervention balanced between groups? | | | | | | | NA |  | | |
|  | 2.6 Was an appropriate analysis used to estimate the effect of assignment to intervention? | | | | | | | Y |  | | |
|  | 2.7 If N/PN/NI to 2.6: Was there potential for a substantial impact (on the result) of the failure to analyse participants in the group to which they were randomized? | | | | | | | NA |  | | |
|  | **Risk of bias judgement** | | | | | | | **Low** |  | | |
| **Bias due to missing outcome data** | 3.1 Were data for this outcome available for all, or nearly all, participants randomized? | | | | | | | Y |  | | |
|  | 3.2 If N/PN/NI to 3.1: Is there evidence that result was not biased by missing outcome data? | | | | | | | NA |  | | |
|  | 3.3 If N/PN to 3.2: Could missingness in the outcome depend on its true value? | | | | | | | NA |  | | |
|  | 3.4 If Y/PY/NI to 3.3: Is it likely that missingness in the outcome depended on its true value? | | | | | | | NA |  |  |  |
|  | **Risk of bias judgement** | | | | | | | **Low** |  | | |
| **Bias in measurement of the outcome** | 4.1 Was the method of measuring the outcome inappropriate? | | | | | | | N |  | | |
|  | 4.2 Could measurement or ascertainment of the outcome have differed between intervention groups? | | | | | | | N |  | | |
|  | 4.3 Were outcome assessors aware of the intervention received by study participants? | | | | | | | N |  | | |
|  | 4.4 If Y/PY/NI to 4.3: Could assessment of the outcome have been influenced by knowledge of intervention received? | | | | | | | NA |  | | |
|  | 4.5 If Y/PY/NI to 4.4: Is it likely that assessment of the outcome was influenced by knowledge of intervention received? | | | | | | | NA |  |  |  |
|  | **Risk of bias judgement** | | | | | | | **Low** |  | | |
| **Bias in selection of the reported result** | 5.1 Were the data that produced this result analysed in accordance with a pre-specified analysis plan that was finalized before unblinded outcome data were available for analysis? | | | | | | | Y |  | | |
|  | 5.2 ... multiple eligible outcome measurements (e.g. scales, definitions, time points) within the outcome domain? | | | | | | | N |  | | |
|  | 5.3 ... multiple eligible analyses of the data? | | | | | | | N |  | | |
|  | **Risk of bias judgement** | | | | | | | **Low** |  | | |
| **Overall bias** | **Risk of bias judgement** | | | | | | | **Low** |  | | |
