# Supplementary material for: Effect of astaxanthin supplementation on female fertility and reproductive outcomes: a systematic review and meta-analysis of clinical and animal studies
Source: J Ovarian Res. 2024 Aug 10;17:163. doi: 10.1186/s13048-024-01472-7 (PMC11316280; doi:10.1186/s13048-024-01472-7)
Supplement: Supplementary file 5 — Supplementary Material 5: Details of Risk of Bias Assessment in Animal Model Studies [file 13048_2024_1472_MOESM5_ESM.docx]

**Supplementary File 5 (S5)**

**Details of Risk of Bias Assessment in Animal Model Studies**

| **Author, Year** | **Description of sources of bias according to reviewers' judgment** |
| --- | --- |
| **Ebrahimi, 2021** | 1. The study does not explain details of randomization procedure or sequence generation, concealment and blinding.  2. It seems that data reporting was incomplete as the results of steroid sex hormones that were mentioned in methods sections, are not reported in the results. also, the study does not report the dose-response curves of AST for the different outcomes or the results of MTT assay in different doses of AST.  3. The study does not specify when the AST and metformin treatments were started and stopped. It seems that they were given during the model induction, not after the model confirmation. This might affect the validity of the PCOS model or the effectiveness of the treatments. |
|  |  |
|  |  |
|  |  |
| **Gunyeli, 2021** | 1. The study does not explain details of the randomization procedure or sequence generation (such as using a computer-generated random number sequence or a random number table), concealment, and blinding.  2. Authors stated that IHC assessments were performed in a blinded manner, while they did not mention blinding for their other assessments.  3. It seems that data reporting was incomplete & outcomes were reported selectively as the results of oxidative stress markers that were mentioned in methods sections, were just reported for ovarian, fallopian, and uterine tissue not for all tissue samples. |
|  |  |
|  |  |
|  |  |
| **Kukurt, 2022** | The study does not mention anything about randomization, sequence generation, concealment, and blinding. |
|  |  |
|  |  |
|  |  |
| **Toktay, 2022** | The study does not explain details of the randomization procedure or sequence generation, concealment, and blinding |
|  |  |
|  |  |
|  |  |
| **Toktay, 2023** | 1. The study does not explain details of the randomization procedure or sequence generation, concealment, and blinding.  2. Some points of study methods is not mentioned in the main manuscript text and are just reported in the abstract like the animal's age or random selection of groups.  3. The study does not specify the detail of interventions like administration route and dissolvent. |
|  |  |
|  |  |
|  |  |
