# Supplementary material for: Effect of astaxanthin supplementation on female fertility and reproductive outcomes: a systematic review and meta-analysis of clinical and animal studies
Source: J Ovarian Res. 2024 Aug 10;17:163. doi: 10.1186/s13048-024-01472-7 (PMC11316280; doi:10.1186/s13048-024-01472-7)
Supplement: Supplementary file 6 — Supplementary Material 6: Study Additional Plots [file 13048_2024_1472_MOESM6_ESM.docx]

**Supplementary File 6 (S6) -** Study Plots


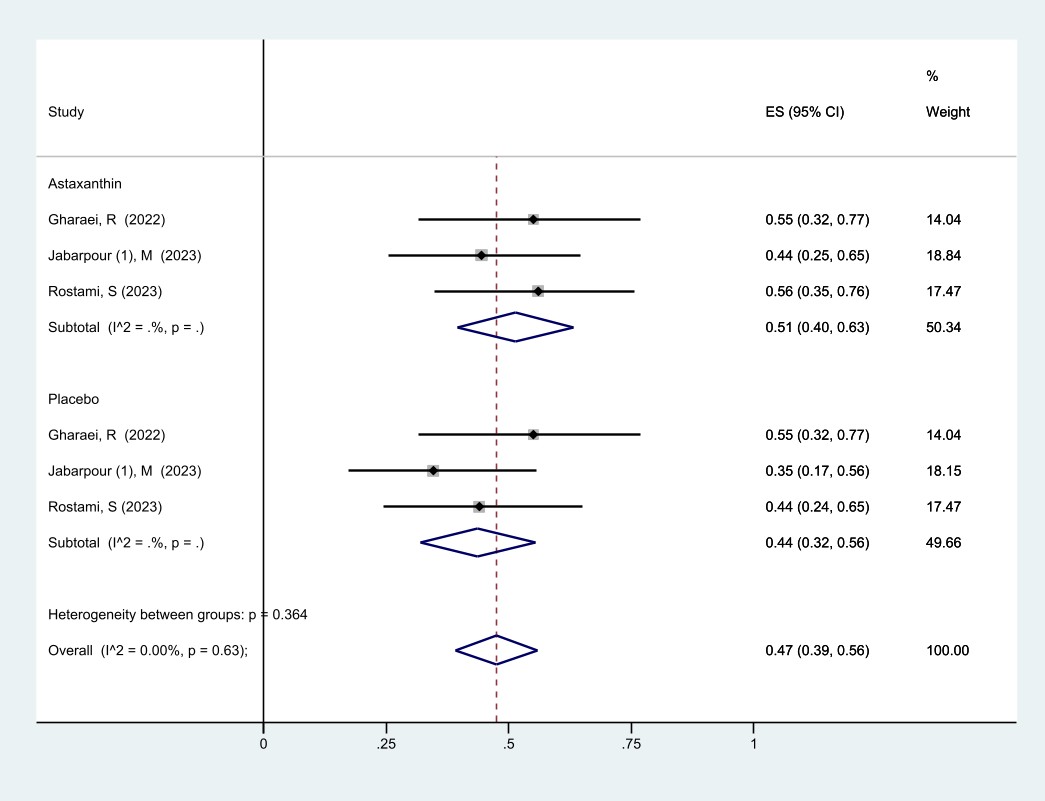


**Plot 1.** Meta-analysis of the effect of Astaxanthin (intervention) compared to the placebo on Pregnancy outcomes (chemical pregnancy rate)


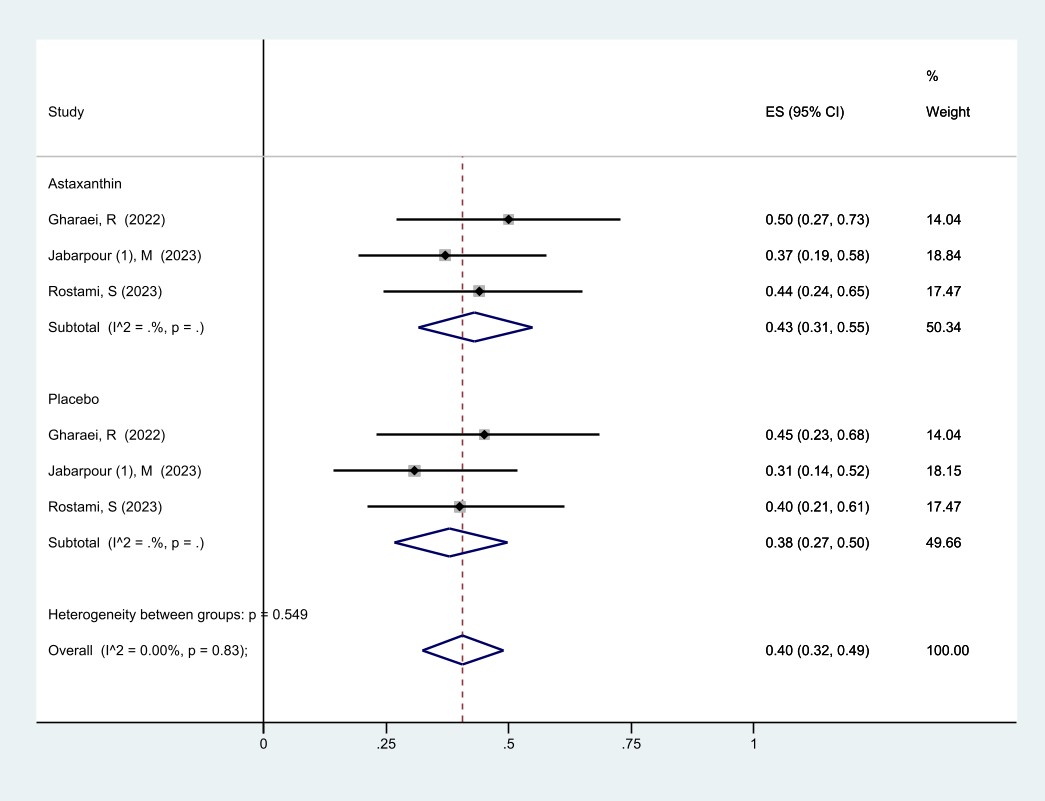


**Plot 2.** Meta-analysis of the effect of Astaxanthin (intervention) compared to the placebo on Pregnancy outcomes (clinical pregnancy rate)
